# Harnessing Pore Size in COF Membranes: A Concentration Gradient-Driven Molecular Dynamics Study on Enhanced H_2_/CH_4_ Separation

**DOI:** 10.1021/acsami.4c20420

**Published:** 2025-03-01

**Authors:** Parivash Jamshidi Ghaleh, Zeynep Pinar Haslak, Merdan Batyrow, Ilknur Erucar

**Affiliations:** † Department of Mechanical Engineering, Faculty of Engineering, Ozyegin University, Cekmekoy, Istanbul 34794, Turkey; ‡ Department of Natural and Mathematical Sciences, Faculty of Engineering, Ozyegin University, Cekmekoy, Istanbul 34794, Turkey

**Keywords:** concentration gradient-driven molecular dynamics, COF membrane, equilibrium molecular dynamics, methane, hydrogen

## Abstract

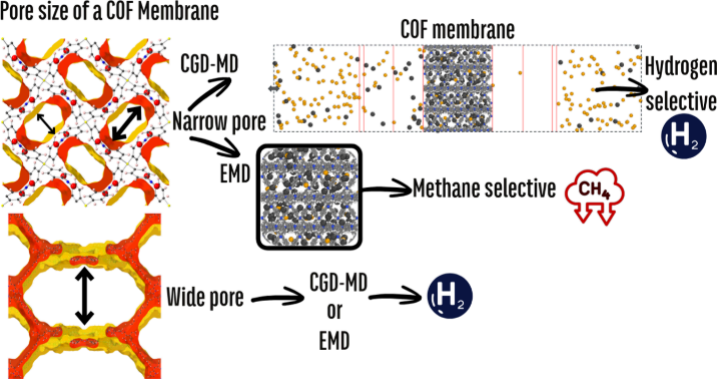

This work presents a novel approach for accurately predicting the gas transport properties of covalent organic framework (COF) membranes using a nonequilibrium molecular dynamics (NEMD) methodology called concentration gradient-driven molecular dynamics (CGD-MD). We first simulated the flux of hydrogen (H_2_) and methane (CH_4_) across two distinct COF membranes, COF-300 and COF-320, for which experimental data are available in the literature. Our CGD-MD simulation results aligned closely with the experimentally measured gas permeability and selectivity of these COF membranes. Leveraging the same methodology, we discovered promising COF candidates for H_2_/CH_4_ separation, including NPN-1, NPN-2, NPN-3, TPE-COF-I, COF-303, DMTA-TPB2, 3D-Por-COF, COF-921, COF-IM AA, TfpBDH, and PCOF-2. We then compared our findings with simulations utilizing the well-known approach that merges grand canonical Monte Carlo (GCMC) and equilibrium molecular dynamics (EMD) to predict gas adsorption and diffusion parameters in COFs. Our results showed that when the pore sizes of COF membranes are below 10 Å, the choice of the method plays a significant role in determining the performance of the membranes. The GCMC+EMD approach suggested that COFs tend to exhibit CH_4_ selectivity when their pore limiting diameters are below 10 Å, whereas the CGD-MD results reveal a preference for H_2_. Density functional theory calculations indicate that H_2_ has a lower affinity for three promising COFs, NPN-1, NPN-2, and NPN-3, compared to CH_4_, which results in H_2_ remaining unbound, while CH_4_ occupies all of the adsorption sites, thereby facilitating the selective recovery of H_2_ at the end of the separation process. We proposed a relationship between adsorption time and diffusion time, highlighting the critical role of selecting an appropriate simulation method. This relationship underscores how adsorption and diffusion processes interplay, impacting material performance. Overall, these insights not only improve the accuracy of predictive models but also guide the development of more efficient COF-based membrane applications for future research and industrial applications.

## 1 Introduction

Hydrogen (H_2_) is known as a crucial energy storage medium in fuel cell systems and as an environmentally friendly substitute for fossil fuels in new generation energy applications. It plays an important role in achieving carbon neutrality and the sustainable development target of future societies by enabling electricity generation using merely water as a byproduct when utilized in fuel cells.^1^

Among various H_2_ production techniques, approximately 96% of H_2_ is generated from fossil fuels, with the steam methane reforming (SMR) method being the most prevalent.^2,3^ The product of the SMR process contains not only H_2_ but also a considerable amount of unreacted methane (CH_4_), indicating the urgent need for the efficient industrial-scale separation of H_2_ and CH_4_. The separation of CH_4_ and H_2_ is indeed a significant challenge due to the chemical and physical resemblance of these gas molecules. Therefore, the industry piles up huge sums of investment to discover new materials that can efficiently and cost-effectively separate these two gases, especially by keeping in mind that H_2_ becomes more crucial by considering the highly set bar of energy transitioning targets of the next coming decades.

Membrane-based H_2_/CH_4_ separation is widely recognized as a practical technology and offers a promising solution with economic and environmental benefits, e.g., convenient fabrication with compact design, low energy consumption, easy operation, and reduced level of waste products.^4,5^ The development of a membrane-based gas separation system requires careful material selection, as the high gas permeability and selectivity of the material are significant for efficient separation. In addition to these properties, the membrane materials should be stable under operating conditions, which requires considerable mechanical strength.^6^ Various membranes, including polymeric materials such as polysulfones and polyimides, inorganic materials such as zeolites, and carbon molecular sieves, have been used for H_2_/CH_4_ separation.^4,7,8^ Among these materials, the performance of polymeric membranes is usually hampered by permeability–selectivity trade-off called the Robeson upper bound,^9^ where an increase in gas permeability leads to a decrease in selectivity and vice versa. On the other hand, inorganic membranes can have superior separation properties; however, they can be considerably expensive and difficult to produce, which limits their practical use.

Among the emerging materials, covalent organic frameworks (COFs)—porous crystalline substances composed of light elements such as boron, silicon, carbon, nitrogen, and oxygen linked by strong covalent bonds^10−13^—are promising membrane materials thanks to their stability, diverse chemical compositions, large surface areas, and high porosities.^14−17^ Gas separation benefits from COFs’ low density in a number of ways, including increased surface area, storage capacity, gas permeability, and energy efficiency; COFs are also cost-effective due to the fact that high gas permeability reduces the membrane area and associated capital cost. These properties make COFs promising materials for practical gas separation applications.^18^ For example, COF-300 was shown to have a high H_2_ permeability of 1.1 × 10^5^ Barrer and an ideal H_2_/CH_4_ selectivity of 3.25.^19^ The COF-320 membrane on porous α-Al_2_O_3_ exhibited a H_2_ permeability of 6.77 × 10^3^ Barrer and an ideal H_2_/CH_4_ selectivity of 2.83.^20^ Fan et al.^21^ synthesized a bilayer COF membrane with a cross-linked pore network, COF-LZU1-ACOF-1, with a very high H_2_/CH_4_ selectivity of 105, outperforming many zeolite imidazolate framework (ZIF) membranes. The same group^22^ later suggested inserting a metal organic framework (MOF) structure inside the COF’s pores via a MOF-in-COF technique to control the size of the COF membrane’s pores. On an α-Al_2_O_3_ substrate, they created a ZIF-67-in-TPPA-1 membrane, which is made up of TPPA-1, 1,3,5-triformylbenzene, and *p*-phenylenediamine. The H_2_/CH_4_ selectivity was 33.3, and the MOF-in-COF membrane showed a H_2_ permeability of up to 3.9 × 10^3^ Barrer, indicating that the MOF-in-COF membrane is one of the best membrane materials for hydrogen separation. They recently synthesized an LA-α-CD-in-TpPa-1 membrane that allows the passage of H_2_ molecules through the transport pathways but limits the diffusion of CH_4_ molecules, boosting the H_2_ permeability (4615.95 Barrer) and H_2_/CH_4_ ideal selectivity (39.4) compared to many zeolites, MOFs, and graphenes.^23^ Recently, composite membranes, COF (TpPa-a) and MXene (Ti_3_C_2_T_*x*_), were fabricated, and a H_2_ permeability of 136.64 Barrer and an ideal H_2_/CH_4_ selectivity of 7.34 were reported.^24^ Using the “MOF-in/on-COF” pore modification strategy, Qi et al.^25^ fabricated ZIF-67-in-Schiff base-based COF membrane (named the PBD membrane), with a high H_2_/CH_4_ selectivity of 33.48. These experiments have demonstrated that COF membranes can improve H_2_/CH_4_ separation technologies, which is promising for their future development.

Given the impracticality of evaluating the performance of a large number of COFs through experimental methods, computational studies are crucial for identifying promising membrane materials. In recent years, computational studies have significantly expanded the literature on the potential of COFs as membrane materials. For example, Tong et al.^26^ computationally demonstrated that COFs, particularly the NPNs (nitroso polymer networks) and COF-1, outperform commonly used zeolites and MOFs in terms of CH_4_/H_2_ adsorption selectivity. Yang et al.^18^ demonstrated that COF-102, -103, -105, and -108 exhibited superior selectivities (11.7, 14, 28.8, and 33.2, respectively) in membrane-based H_2_/CH_4_ separation at 10 bar and 298 K, outperforming widely studied MOFs, IRMOF-1, and Cu-BTC. Keskin’s group^27^ showed the potential of COFs as promising membrane materials for separation of H_2_ from CH_4_, and by analyzing 572 COFs, they revealed that several COFs with large porosities showed a good combination of H_2_ permeability (>10^5^ Barrer) and H_2_/CH_4_ selectivities up to 4.6 at 1 bar and 298 K.^28^ The same group also investigated 589 experimentally synthesized COFs and 120 hypothetical (computer-generated) COFs to assess their potential in He/CH_4_, He/N_2_, H_2_/CH_4_, and H_2_/N_2_ separations.^29^ COFs with high pore limiting diameters (PLDs) (ranging from 18 to 45 Å) were shown to be the most promising for H_2_/CH_4_ separation, achieving a maximum selectivity of 6.3. In all these studies, the gas permeability data were obtained from the results of equilibrium molecular dynamics (EMD) simulations in conjunction with grand canonical Monte Carlo (GCMC) simulations. However, recent studies have shown that the use of self-diffusion coefficients derived from EMD simulations to calculate gas transport properties can lead to inaccurate values for permselectivity^30^ due to inherent assumptions within this approach. When gas molecules do not interact strongly, as they do at low pressures, the GCMC+EMD method, which is based on the solution–diffusion model, is anticipated to be more reliable.^30^ In addition, the GCMC+EMD method ignores the possibility of mass transfer resistance at the membrane surface in favor of simulations of crystal structures performed under periodic boundary conditions (PBCs).

The nonequilibrium molecular dynamics (NEMD) method offers a unique advantage in predicting gas transport through nanoporous membranes, as gas permeabilities are calculated directly from the simulation setup. In contrast to EMD simulations, NEMD simulations take into account the mass transfer resistance and the pore entrance resistance, which enables a more accurate representation of the actual mass transfer phenomena. This approach enables accurate assessment of gas permeation through membranes under various conditions, including concentration gradients, and provides valuable insight into membrane performance and design optimization.^31,32^ For example, Velioglu and Keskin^33^ conducted a comparative study between a conventional NEMD approach and the GCMC+EMD method, focusing on the permeation of H_2_ and CH_4_ in several well-known MOF membranes. Their analysis showed that the GCMC+EMD method consistently overestimates the permeability of H_2_ and CH_4_ compared to experimental measurements. While the NEMD simulations showed good agreement with the experimental data for H_2_/CH_4_ permeability and selectivity, for certain membranes such as ZIF-8, the GCMC+EMD method was observed to have an inverse selectivity in favor of CH_4_ over H_2_, as the estimated permeability is higher for CH_4_. This method uses an impermeable, movable wall on one side of the membrane to create a pressure gradient and drive the molecular motion. However, it proved to be unsuitable for steady-state membrane transport and mixture gas separation simulations, as it leads to feed depletion, and the composition of the feed cannot be controlled during the simulation. A further investigation of NEMD was later carried out to investigate the potential of MOF membranes in separation of H_2_/CH_4_ gases, in which a dual-force zone nonequilibrium MD technique (DFZ-NEMD) was used for both functionalized and unfunctionalized IRMOF-1 structures.^34^ The results obtained from single-component DFZ-NEMD simulations of H_2_ and CH_4_ with permeability values of 2.59 × 10^5^ Barrer and 0.69 × 10^5^ Barrer, respectively, showed good agreement with experimental results. Ozcan et al.^35^ introduced an enhanced simulation approach, termed concentration gradient-driven molecular dynamics (CGD-MD), which provides higher precision by regulating the fluid concentrations through the application of self-regulating bias forces on both the feed and permeate sides of the membrane. In contrast to other NEMD methods, in which the fluid molecules are forced to diffuse through the membrane, molecular diffusion is not forced by external forces on individual molecules in this approach. The CGD-MD method has been successfully used in evaluating the permselectivity of ZIF-8^30^ and MOF-polymer mixed matrix membranes.^36^ In a recent work,^37^ the gas transport through MXene nanopores was examined using NEMD simulations. The results demonstrated that the mechanism of transport of CH_4_ through MXene nanopores changes when the pore size increases above a threshold value, which is 3–4 Å for CH_4_. There are primarily two ways, in which gas molecules can pass through MXene nanopores, depending on the pore size: first, when pore size of the MXene ≤ pore size of the gas, the gas molecules pass through via molecular sieving; second, when pore size of the MXene > pore size of the gas, the gas molecules pass through via Knudsen diffusion.

Motivated by this work, we performed NEMD simulations to analyze gas transport through COF membranes for the first time in the literature, providing realistic insight into their gas transport behavior. By applying the CGD-MD method to several COF membranes, we aim to gain a comprehensive understanding of the complicated diffusion and separation mechanisms of H_2_/CH_4_ gas mixtures. Initially, we focused on the validation of our method using existing experimental data with COF-300 and COF-320, which have already been fabricated and tested.^19,20^ The permeation selectivities predicted with the CGD-MD method were systematically compared with those obtained with the widely used GCMC+EMD approach^28,29^ for 13 different COF membranes for H_2_/CH_4_ separation. Finally, we performed density functional theory (DFT) calculations on three promising COF membranes with high H_2_/CH_4_ selectivity to better understand the gas transport mechanism.

## 2 Computational Methods and Details

**Figure 1:**
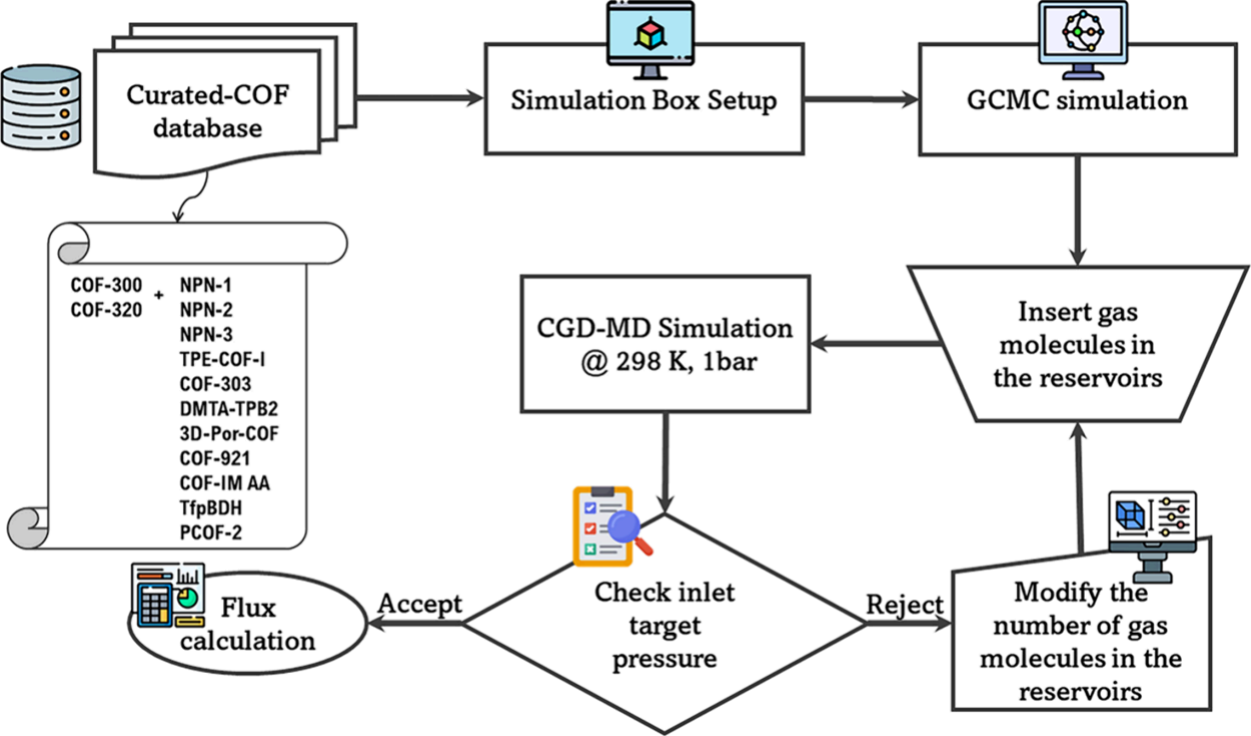
Schematic representation of the computational methodology used in this work.

Figure 1 depicts our computational procedure to systematically assess the gas permeation and separation performance of the COF membranes. We used the concentration gradient-driven molecular dynamics (CGD-MD) method to accurately represent an experimental membrane system and simulate the steady-state mass flow of H_2_ and CH_4_ through 13 distinct COF membranes. Detailed information on simulation box sizes and physical and chemical properties of the COF structures used in this study is provided in Table S1 of the Supporting Information (SI).

Zeo++ software (version 0.3)^38^ was used to compute geometry-based characteristics of COFs, including PLDs, the largest cavity diameter (LCD), accessible surface area (ASA), and accessible pore volume fraction. A probe radius equivalent to 1.86 Å, representing the kinetic radius of N_2_, and 2 × 10^3^ Monte Carlo steps were used for predicting the surface area. The accessible volume fraction was determined by setting a ghost probe and 5 × 10^4^ Monte Carlo steps.

We first computed single-component H_2_ and CH_4_ flux on COF-300 and COF-320 to validate the method with experimental results. We used the CURATED-COF^39^ database to obtain the crystallographic coordinates of COFs, including original and optimized structures. To model the supercells, the unit cells of COF structures were expanded using the LAMMPS-interface package.^40^ For example, COF-300 was replicated in a 2 × 2 × 5 configuration in the *x*, *y*, and *z* directions, respectively. This was done to achieve a reasonable size and ensure that enough particles were present to accommodate the guest molecules responsible for creating pressure on the surface of the membrane. We employed the universal force field (UFF)^41^ for Lennard-Jones (LJ) 12-6 potential parameters of COF atoms. A test run was conducted with the DREIDING force field^42^ for COF-300 to validate the findings obtained with the UFF.

**Figure 2:**
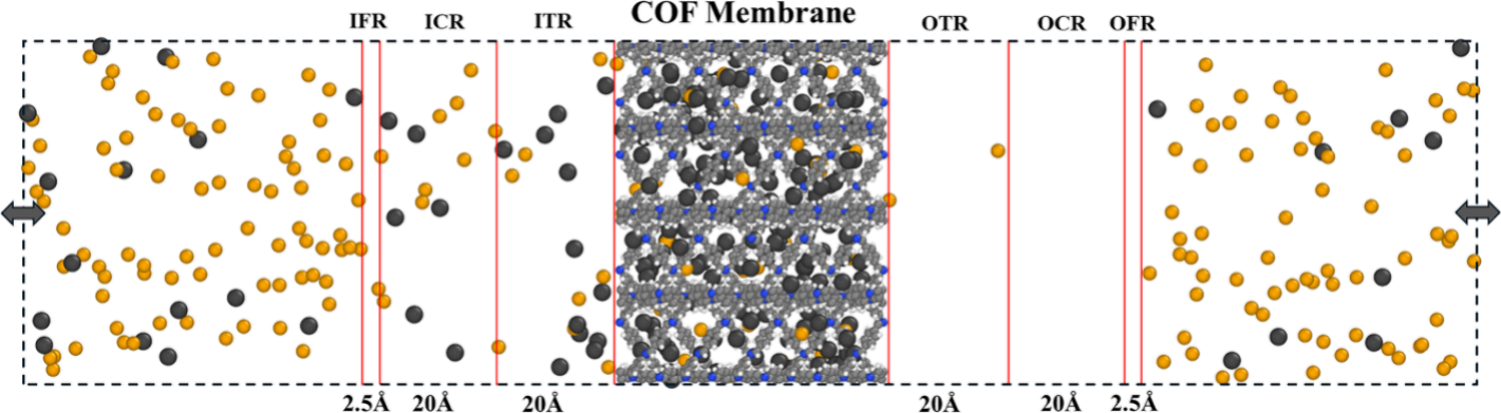
Representation of the simulation system in the CGD-MD method, where fluid molecules exhibit unrestricted movement between opposing sides, owing to the PBC. The dashed lines represent the simulation box. Within the COF membrane, carbon, nitrogen, and hydrogen atoms are depicted in gray, blue, and white, respectively. H_2_ and CH_4_ molecules are colored orange and black, respectively.

Figure 2 shows a schematic representation of the membrane system used in CGD-MD simulations. The membrane was positioned at the center of a simulation box, with dimensions matching the membrane size in the *x* and *y* directions, while extending 100 Å in the *z*-direction from each side. In NEMD simulations, external forces allow for the creation of controlled concentration gradients, which are essential for studying diffusion and transport mechanisms under nonequilibrium behavior. The inlet control region (ICR) and outlet control region (OCR) are located at the feed and permeate sides of the membrane, respectively. These control regions are separated from the membrane by two transition zones: the ITR and the OTR. To regulate the concentrations within the ICR and the OCR, two different external forces, which are defined in eqs 1 and 2, were applied to the fluid molecules within the separate inlet force region (IFR) and outlet force region (OFR), situated adjacent to the ICR and the OCR, respectively. These forces act perpendicular to the membrane surface, adjusting the continuous molecular flux through the IFR (and OFR) to maintain the concentration within the ICR (and OCR) at predetermined target values. This configuration enables the establishment of different concentrations in the inlet and outlet control regions.^35,43,44^
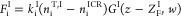
1
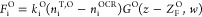
2

In eqs 1 and 2, *k*_i_^I^ and *k*_i_^O^ stand for the force constants (15,000 and 50,000 kJ·nm^3^·mol^–1^, respectively), which ensure that the fluid concentration in the ICR and OCR remains close to the target values (*n*_i_^T, I^ and *n*_i_^T, O^) by adjusting the magnitude of the force *F*_i_^I^ and *F*_i_^O^ based on the deviation from the target concentrations. *n*_i_^ICR^ and *n*_i_^OCR^ are the instantaneous concentrations in the ICR and OCR, respectively. *G*^I^ and *G*^O^ are bell-shaped functions characterized by a width *w* and centered at *Z*_F_^I^ and *Z*_F_^O^, which represent the *z*-coordinates of the IFR and OFR, respectively.^35^

The initial determination of the number of gas molecules required for the simulation setup was carried out using an estimation process based on the Grand Canonical Monte Carlo (GCMC) ensemble applied to the COF structures. To apply this procedure, we performed GCMC simulations at 298 K and a pressure equivalent to the average of the target pressures within the ICR and OCR, using the RASPA2 simulation package.^45^ The number of molecules was incrementally adjusted to achieve the desired pressure within the specified control regions. The CH_4_ and H_2_ molecules were modeled using the united atom representation from the LJ potential using the TraPPE force fields with CH_4_ (ε = 0.294 kcal/mol, σ = 3.73 Å)^46^ and H_2_ (ε = 0.068 kcal/mol, σ = 2.96 Å).^47^ TraPPE is a specialized and transferable force field meticulously parametrized to accurately describe the thermophysical properties of gas molecules. The combination of UFF and TraPPE force fields has demonstrated strong agreement between simulated and experimental isotherms of COFs for various adsorbate molecules.^29,39^ Packmol simulation package^48^ version 20.14.2 was then used to generate the initial positions of gas molecules in the simulation box. The gas molecules were randomly distributed along the *z*-direction, specifically confined to a region spanning 0–90 Å while occupying the full dimensions of the simulation box in the *x* and *y* directions. Following this initial configuration, an *NVT* ensemble simulation was performed to equilibrate the system, promoting a uniform distribution of the gas molecules throughout the simulation box.

CGD-MD simulations were conducted using the Large-scale Atomic/Molecular Massively Parallel Simulator (LAMMPS)^49^ software with the *NVT* ensemble framework. To account for short-range van der Waals interactions, the LJ potential was used, employing a cutoff distance of 12.5 Å. The temperature was adjusted using the Nosé–Hoover thermostat to hold constant at 298 K, and two separate thermostats were used for each gas in the mixture simulations to prevent asymmetric thermalization due to hot solute–cold solvent effect.^50^ PBCs were applied in all directions. A time step of 1 fs was used, and simulations were equilibrated for 2 ns, followed by a production run for a duration of 20 ns. Each simulation was repeated three times to calculate the error bars. Atomistic trajectories were recorded at 1 ps intervals for subsequent analysis of the flux and density profiles. The biased forces were applied using a modified version of the PLUMED 2.9.0 plugin.^51^ In the ICR, we aimed to achieve a concentration corresponding to the total feed pressure of 1 bar, derived from the pressure/density data of the NIST database.^52^ For the OCR, we aimed to achieve a concentration of 0 to create a vacuum effect on the permeate side in all our simulations. The widths of the ICR, the OCR, the ITR, and the OTR were maintained at 20 Å, while the widths of the IFR and OFR were set at 2.5 Å. More details of the computational approach to bias forces and the classification of pressure/concentration difference control region are provided by Ozcan et al.^35^

The measurement of H_2_ and CH_4_ flux through a COF membrane was determined by counting the net number of gas molecules (H_2_ or CH_4_) that cross a *xy*-plane within the membrane. This plane is aligned orthogonally to the *z*-direction and located at the center of the membrane. By dividing this net molecule count by the product of the cross-sectional area (*A*_*xy*_) and the time interval (*t*), we obtain the gas flux (*J*_*z*_). The formula for gas flux is expressed as
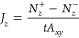
3where *N*_*z*_^+^ and *N*_*z*_^–^ represent the number of molecules (H_2_ or CH_4_) crossing the *xy*-plane in the positive and negative *z*-direction, respectively. As an example, the molecule passage as a function of time during a simulation is presented in Figure S1.

The permeability (Π) of H_2_ and CH_4_ was calculated by the equation
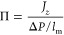
4where Δ*P* is the fluid’s pressure difference between ICR and OCR regions, which was estimated through the NIST database,^52^ corresponding to the concentration difference of these two regions, and *l*_m_ denotes membrane thickness in the *z*-direction.

Selectivity of H_2_ over CH_4_ was calculated according to the following formula:
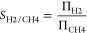
5

This formula was applied to both single and mixed gases to determine the ideal selectivity and the mixture selectivity.

For further investigation of the interactions of CH_4_ and H_2_ with three selected COFs (NPN-1, NPN-2, NPN-3), DFT calculations were performed on cluster models, as shown in Figure S2, which represent the linkers of the COFs. The connecting carbon atoms of phenyl groups on linkers were kept frozen in their crystalline positions during the geometry optimization procedure to mimic the constrained COF environment, while the rest of the atoms were fully optimized. Geometry optimization of all possible COF–gas pair conformations was performed using the Becke three-parameter Lee–Yang–Parr (B3LYP)^53,54^ functional with D2 correction scheme proposed by Grimme et al.^55^ and all-electron 6-31+G* basis set using the Gaussian16 program package.^56^ Vibrational frequency analysis was performed to ensure that there is no imaginary frequency. The binding energies (Δ*E*_bind_) between CH_4_/H_2_ and the COFs were calculated by using eq 6:
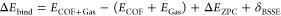
6

in which the energy of the COF–gas complex is represented by *E*_COF+Gas_, the energies of COF and gas are represented by *E*_COF_ and *E*_Gas_, the zero-point energy correction term of the system is denoted by Δ*E*_ZPC_, and δ_BSSE_ is the basis set superposition error correction employed by the Counter Poise approach.

## 3 Results and Discussion

**Figure 3:**
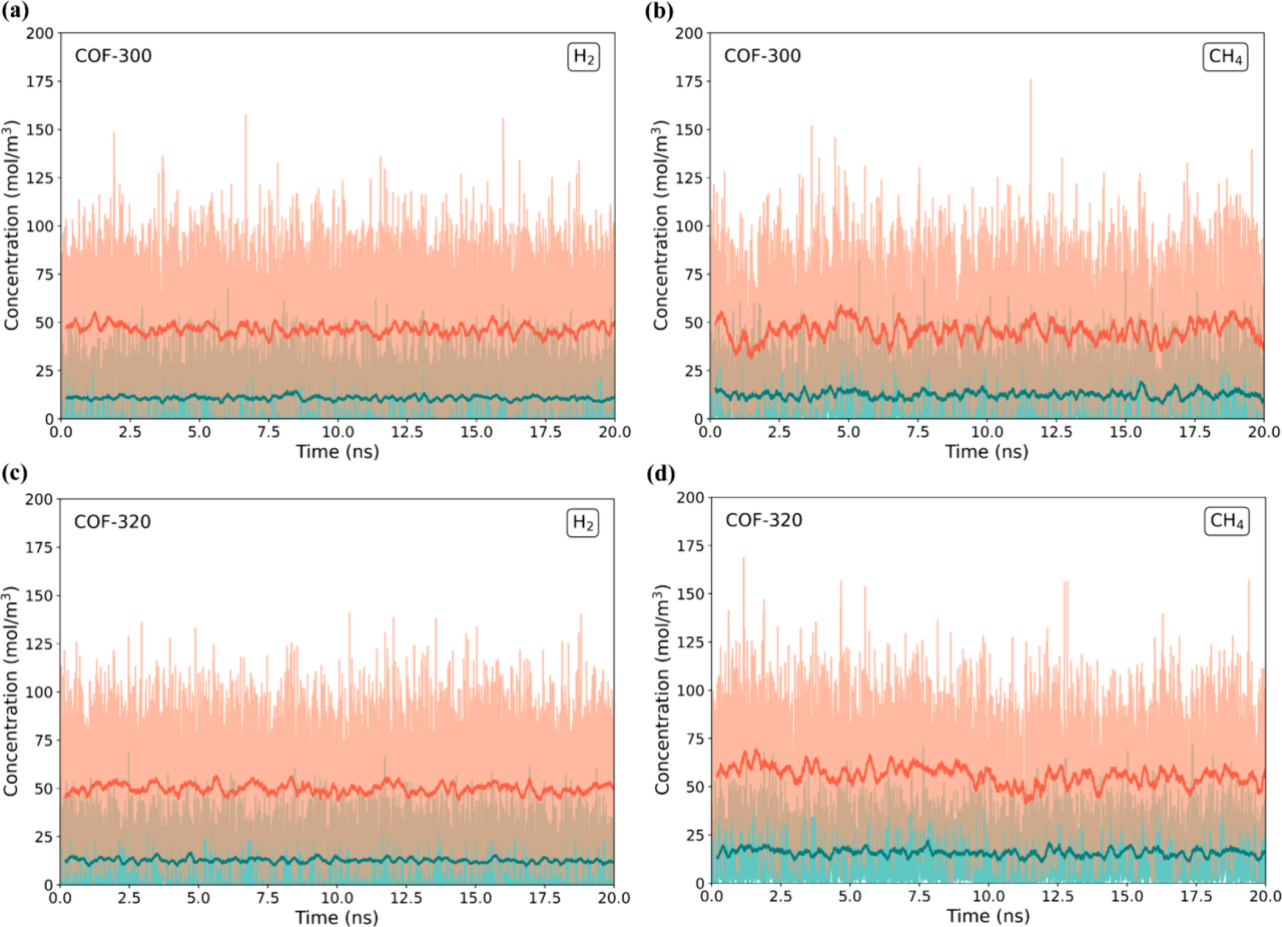
Inlet and outlet concentrations for (a) H_2_ in COF-300, (b) CH_4_ in COF-300, (c) H_2_ in COF-320, and (d) CH_4_ in COF-320 as a function of time in production runs. The momentary values of ICR and OCR concentration are depicted in light orange and light green, respectively, while their moving averages, calculated with a smoothing time of 0.2 ns, are represented by solid lines of the same colors.

Figure 3 illustrates the concentration profiles in both the ICR and the OCR during the production run for pure H_2_ and CH_4_ as a function of simulation time for the COF-300 and COF-320 membranes. The gas concentrations in both regions remained stable throughout the simulation, fluctuating only around their average values, confirming that the system had reached an equilibrium condition.

Similarly, the concentration profiles of NPN-1, NPN-2, NPN-3, TPE-COF-I, COF-300 (mixture gas profile), COF-303, DMTA-TPB2, 3D-Por-COF, COF-921, COF-IM AA, TfpBDH, and PCOF-2, as shown in Figures S3–S6, remained stable during the entire simulations. Figure S7 presents the *z*-direction density profiles of CH_4_ and H_2_ across the membrane in the simulation box, illustrating the concentration gradient through the membrane. These profiles were computed by averaging the number of gas molecules in 0.1 nm bins along the *z*-direction and dividing the 20 ns production run into five equal time intervals to calculate error bars. Statistical analysis revealed negligible deviations, further confirming the equilibrium conditions and enabling accurate flux calculations. The observed density peaks correspond to adsorbed gas molecules within the COF membrane pores, with higher peaks for CH_4_, indicating a preferential adsorption of CH_4_ over H_2_ in the COF membranes. Overall, the consistency of the gas concentration profiles and the small fluctuations in density profiles confirm that the system reached equilibrium, providing a reliable foundation for interpreting the computational results.

**Figure 4:**
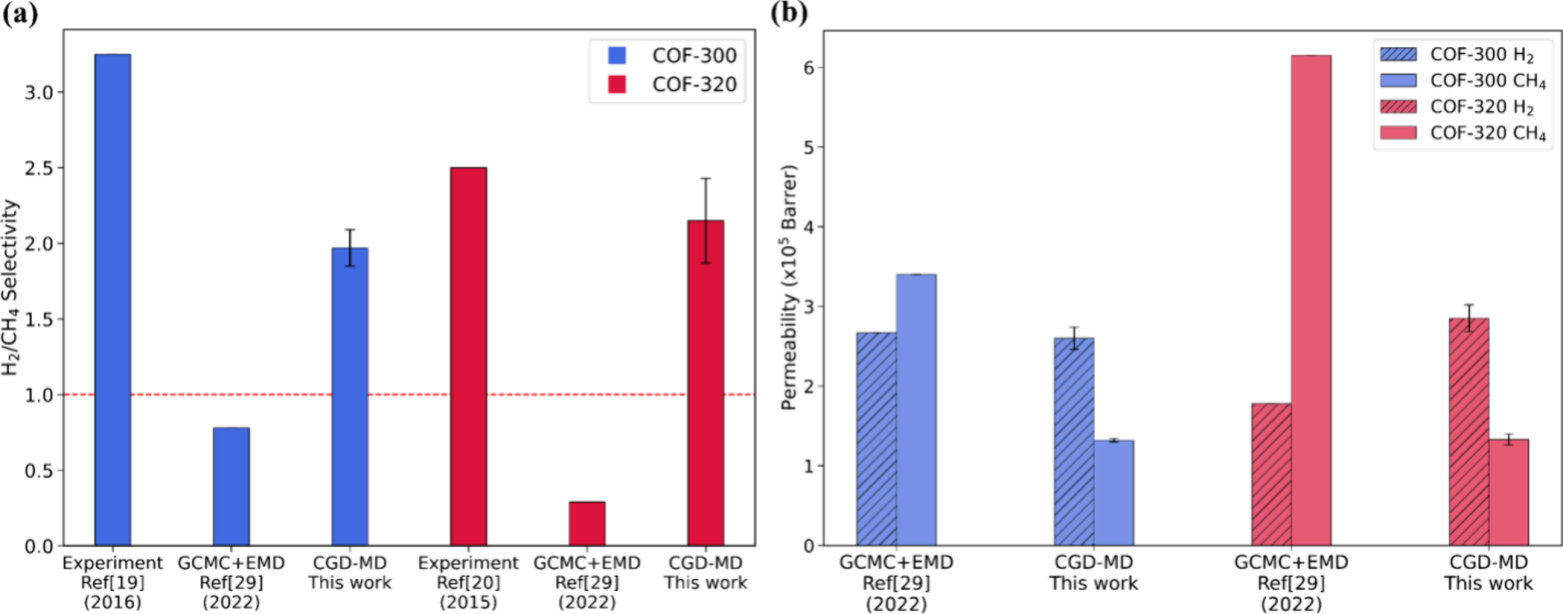
Comparison of (a) ideal selectivities of experimental and simulated COF-300 and COF-320 and (b) permeabilities of COF-300 and COF-320 by GCMC+EMD and CGD-MD methods.

We compared the results from our CGD-MD simulations with the available experimental data for the synthesis of COF-300^19^ and COF-320^20^ at 298 K and 1 bar. Figure 4a presents a comparison of the ideal permselectivities of COF-300 and COF-320, computed by using both the CGD-MD and GCMC+EMD methods, against experimental measurements for H_2_/CH_4_ separation.

As shown in Figure 4a, the GCMC+EMD results reveal that both COF-300 and COF-320 exhibit CH_4_ selectivity over H_2_, with ideal selectivity values of 0.78 and 0.29, respectively. Conversely, the H_2_/CH_4_ ideal permselectivities derived from the CGD-MD simulations were 1.97 ± 0.12 for COF-300 and 2.15 ± 0.28 for COF-320, demonstrating good agreement with the experimental values of 3.25^19^ and 2.52,^20^ respectively. To assess the sensitivity of our results to different force-field parameters, we also performed three independent CGD-MD simulations for COF-300 using the DREIDING force field. The computed concentration profiles for the ICR and OCR are presented in Figure S8. The calculated H_2_/CH_4_ ideal permselectivities are found to be 2.25 ± 0.02, as presented in Table S2, which is also in harmony with the experimental findings. These findings underscore the reliability of the CGD-MD method in generating accurate data for qualitative analyses.

Figure 4b further clarifies the reverse selectivity for CH_4_ over H_2_ observed in the GCMC+EMD method. The overprediction of CH_4_ permeability in this method may be attributed to the use of the self-diffusion coefficient, which is typically higher than the transport diffusivity.^31,57^ The self-diffusion coefficients of CH_4_ have been reported as 1.5 × 10^–4^ cm^2^/s for COF-300 and 1.48 × 10^–4^ cm^2^/s for COF-320, while the CH_4_ uptake values were 1.51 and 1.37 mol/kg, respectively. Additionally, the GCMC+EMD method does not consider bulk gas-phase transport and surface resistance effects on the feed side of the membrane, which could explain the discrepancy in the selectivity results.

In addition to the single-component gas membrane system, we performed simulations of COF-300 for a H_2_/CH_4_ mixture with a composition of 20 H_2_ and 45 CH_4_ molecules. The predicted permeability values of COF-300 for single-component H_2_ and CH_4_ were 2.6 ± 0.14 × 10^5^ and 1.32 ± 0.02 × 10^5^ Barrer, respectively. The higher permeability of H_2_ compared to CH_4_ is expected since the molecules with smaller kinetic diameters and molar masses tend to diffuse faster through the membrane compared to ones with larger kinetic diameters and molar masses (*d*_H_2__ = 2.9 vs *d*_CH_4__ = 3.8 Å). However, when the gases were present together in the mixture, the permeabilities slightly decreased to 2.26 ± 0.09 × 10^5^ Barrer for H_2_ and 1.17 ± 0.12 × 10^5^ Barrer for CH_4_. This reduction was attributed to competitive adsorption between the molecules and spatial hindrance within the membrane pores. Notably, the decrease in H_2_ permeability is more pronounced than that of CH_4_, which can be explained by the pore blockage caused by competitively adsorbed CH_4_ molecules, as shown in Figure S9. During single-component gas simulations, the pores are exclusively accessible to H_2_ molecules.^36^ However, the presence of H_2_ molecules in the mixture disrupts the efficient packing of CH_4_ within the pores, slightly reducing the CH_4_ permeability. Figure S10 represents the mean residence time analyses for both single-component and mixture gas simulations through the COF-300 membrane. As shown in Figure S10, the mean residence time of both gases decreases slightly in the mixture compared to single-component gas simulations. Unlike H_2_, the residence time profile of CH_4_ in the membrane exhibits large variations due to its relatively stronger adsorption in the structure. Consequently, CH_4_ spends more time in the membrane than H_2_, further emphasizing the competitive interactions between the two gases.

**Figure 5:**
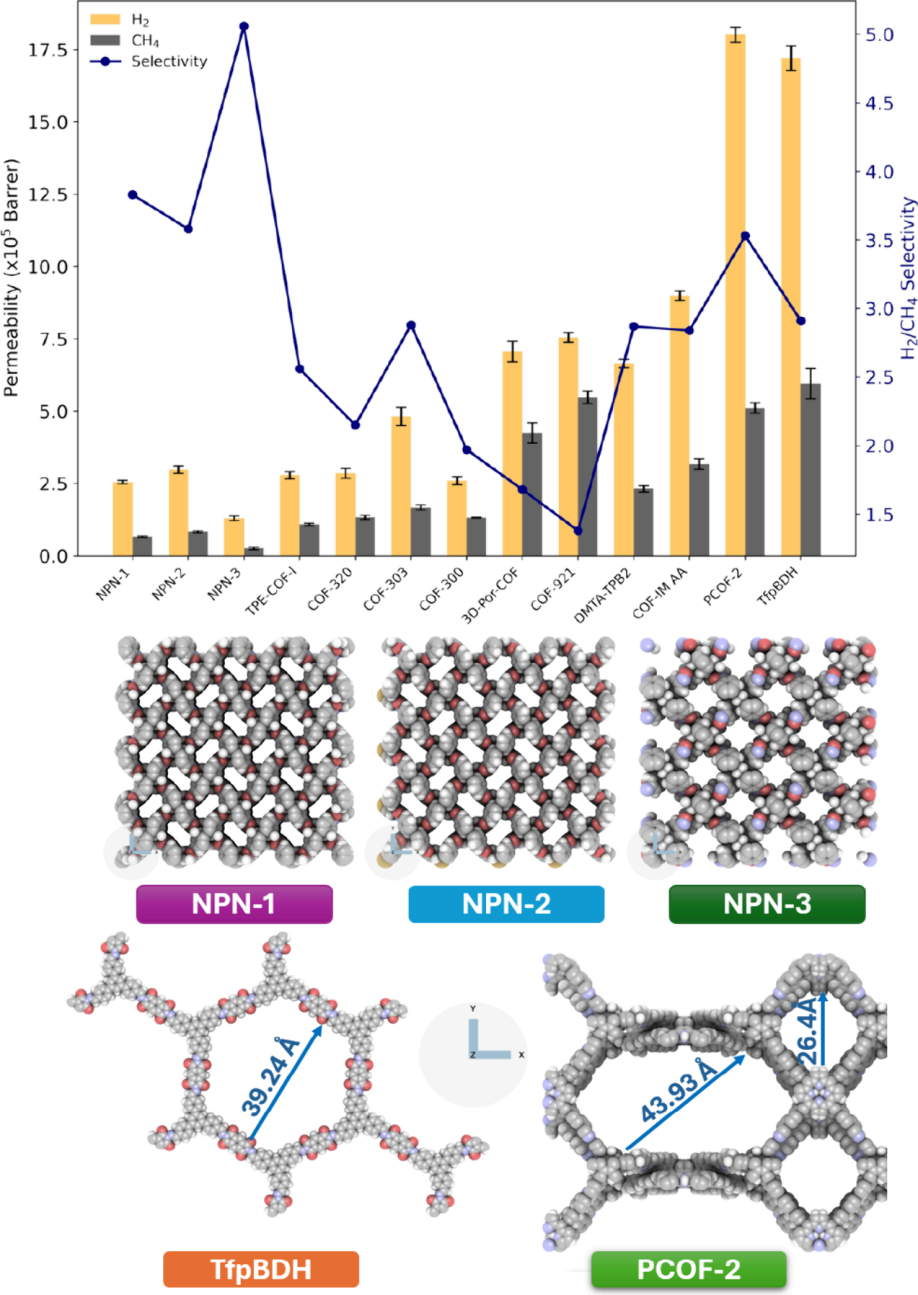
CGD-MD simulation results of 13 different COF membranes (top) and schematic of the best H_2_/CH_4_ selective COF membranes (bottom). The colors representing carbon, nitrogen, hydrogen, oxygen, and silicon atoms are gray, blue, white, red, and yellow, respectively.

To further investigate the discrepancies observed in the GCM+EMD method, we examined the potential of 11 additional COF membranes (excluding COF-320 and COF-320) with PLDs ranging from 4 to 26 Å. As shown in Figure 5, we applied the CGD-MD method to a total of 13 COF structures to assess their H_2_ and CH_4_ permeation selectivities at 298 K and 1 bar. The results indicated that all 13 COF structures exhibited H_2_ permeation higher than that of CH_4_, as expected. The COF structures, arranged by increasing the PLD from left to right in Figure 5, reveal a trend of increasing H_2_ permeability with larger PLDs. However, H_2_/CH_4_ selectivity did not show a corresponding increase with larger pore sizes.

**Figure 6:**
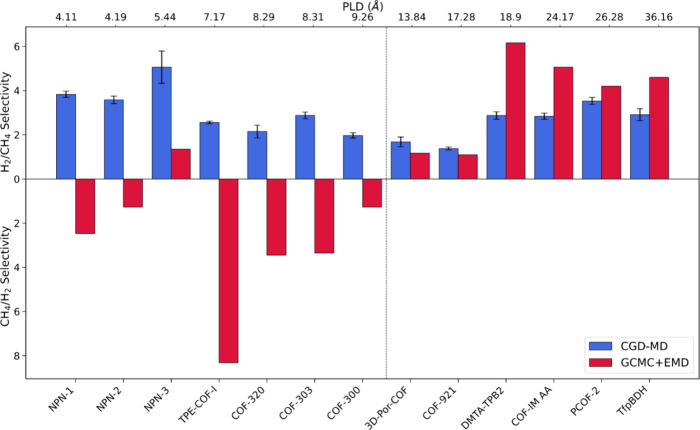
Comparison of the CGD-MD and GCMC+EMD methods for structures with various PLD ranges. Values above the *x*-axis represent the H_2_/CH_4_ ideal selectivity for simulations with a higher H_2_ permeability than CH_4_. Values below the *x*-axis represent the CH_4_/H_2_ ideal selectivity for simulations indicating higher CH_4_ permeability than H_2_.

Figure 6 provides a comparative analysis of our findings with those from GCMC+EMD simulations reported by Keskin and co-workers.^29^ Their results suggested that COF structures with PLDs smaller than approximately 10 Å are more likely to favor CH_4_ selectivity over H_2_, which contrasts with both our predictions and the experimental findings^19,20^ in the literature. These studies consistently demonstrate higher H_2_ selectivity over CH_4_ in the COF structures, even with smaller pore sizes. This suggests that the GCMC+EMD method may underestimate the performance of COFs for H_2_/CH_4_ separation, likely due to limitations in accounting for collective transport, surface resistance, and respective filling of gas molecules inside pores. Our method that we implement in this study, in contrast, appears to offer a more accurate understanding of gas permeation behavior across a wider range of pore sizes.

**Figure 7:**
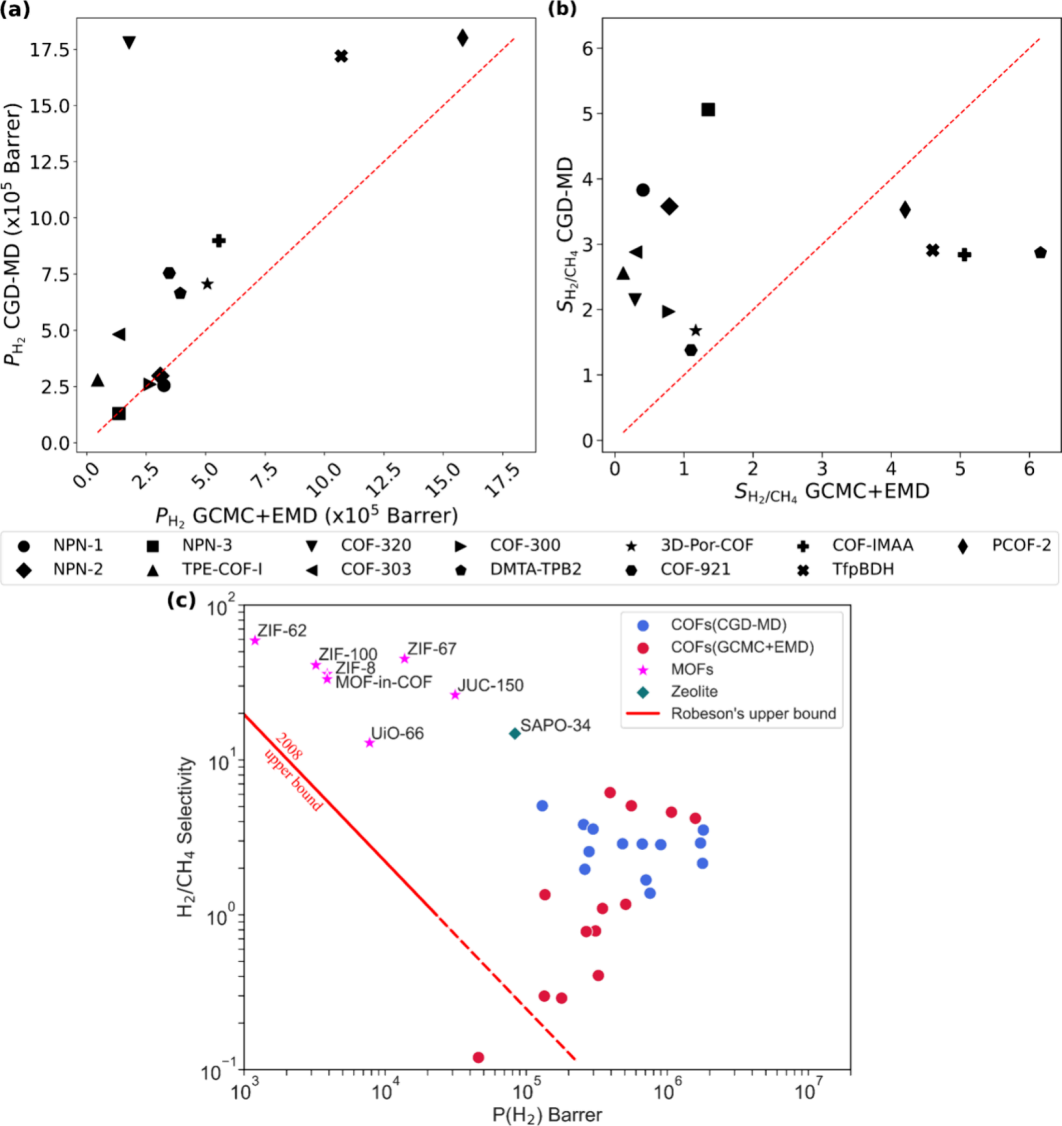
Comparative analysis of (a) H_2_ permeability and (b) H_2_/CH_4_ ideal selectivity of 13 COFs based on our CGD-MD simulations and the previously reported^29^ GCMC+EMD method. (c) H_2_ permeabilities and H_2_/CH_4_ selectivities of 13 COFs, along with experimentally reported promising MOFs (ZIF-62, ZIF-100, ZIF-8, ZIF-67, JUC-150, UiO-66, and MOF-in-COF)^22,59−63^ and a zeolite (SAPO-34),^64^ at 1 bar and 298 K, plotted against Robeson’s upper bound.^9^

The comparison of H_2_ permeabilities (Figure 7a) and H_2_/CH_4_ permeation selectivities (Figure 7b) of the CGD-MD method as a function of the GCMC+EMD method of the 13 COF membranes is presented. For comparison, Robeson’s upper bound,^9^ representing the performance limit of polymeric membranes, is included in Figure 7c, alongside promising MOF membranes (ZIF-62, ZIF-100, ZIF-8, ZIF-67, JUC-150, Uio-66, MOF-in-COF) and a Zeolite named SAPO-34 under the same pressure and temperature conditions. Notably, TPE-COF-I, which was previously found to be below the performance limit using GCMC+EMD, surpassed the upper bound with CGD-MD, achieving a selectivity of 2.56 ± 0.06 and a H_2_ permeability of 2.79 ± 0.13 × 10^5^ Barrer. Among the COFs examined, NPN-3 emerged as the best performer for H_2_/CH_4_ separation, with a selectivity of 5.06 ± 0.73 and a H_2_ permeability of 1.3 ± 0.08 × 10^5^ Barrer. To investigate the performance of NPN-3 at higher pressure, we also performed our CGD-MD simulations at 10 bar (as a feed pressure) and 298 K, for which the concentration profiles for the ICR and OCR are displayed in Figure S11. Under these conditions, the H_2_/CH_4_ selectivity of NPN-3 significantly increased to 201, while the H_2_ permeability decreased slightly to 0.94 × 10^5^ Barrer. Tong et al. simulated the H_2_/CH_4_ selectivity of various restacked COF-6 membranes and found a maximum selectivity of 81.1 at 10 bar, which is almost three times lower than that of NPN-3.^58^

All the COFs in this study demonstrated superior performance compared to polymeric membranes, as indicated by their positioning above the Robeson’s upper bound.^9^ The large pore sizes of the COFs enable them to achieve significantly higher H_2_ permeabilities. However, H_2_/CH_4_ selectivities were lower compared to those of MOFs, indicating that while COFs exhibit exceptional permeability, their selectivity for H_2_ over CH_4_ remains limited. This trade-off between permeability and selectivity is particularly evident when compared with MOFs, which offer a more balanced performance for H_2_/CH_4_ separation. Consequently, while COFs show great promise for gas separation applications, further optimization is needed to improve their selectivity in order to rival the overall separation efficiency of MOFs.

**Figure 8:**
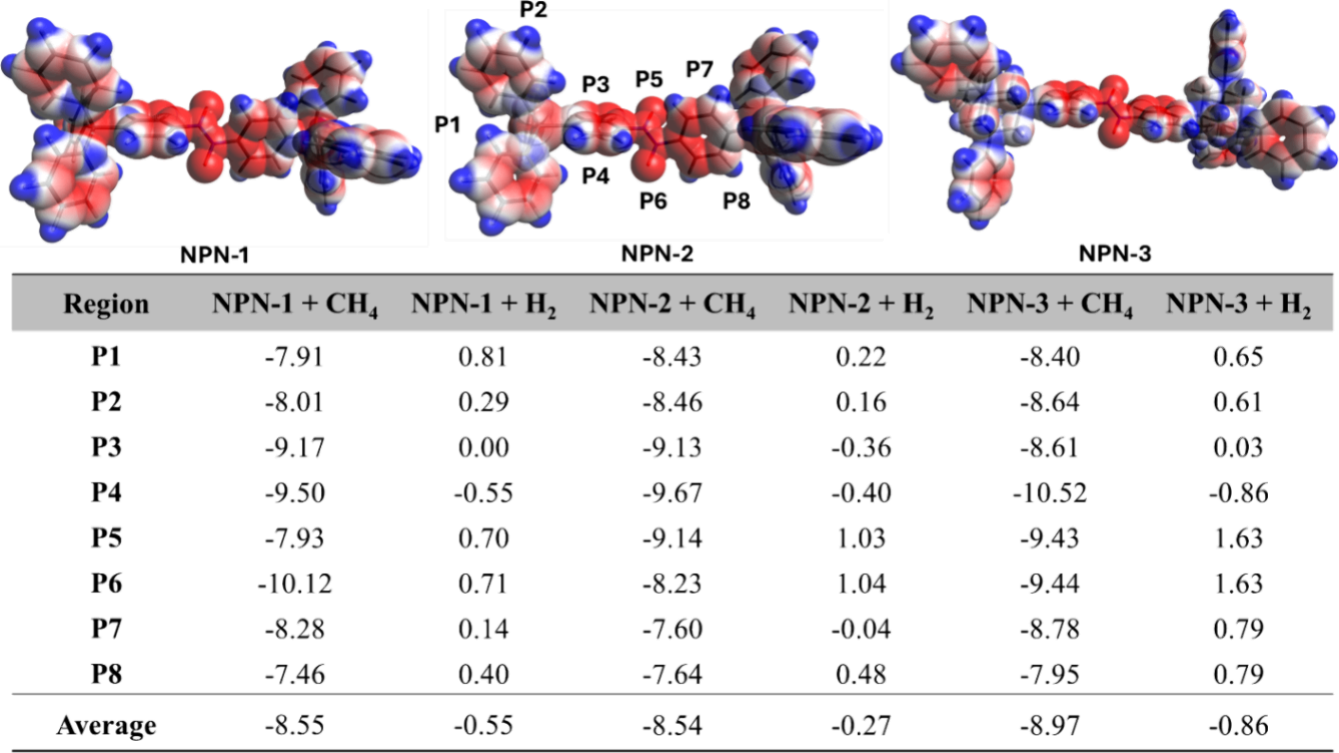
Calculated binding energies (Δ*E*_bind_, kJ/mol) of the COF–gas pairs with respect to the positions of the gases.

We performed DFT calculations to gain deeper insights into the gas adsorption and diffusion mechanisms in three selected COFs: NPN-1, NPN-2, and NPN-3. These COFs are composed of azodioxy-linked tetraphenylmethane (NPN-1), tetraphenylsilane (NPN-2), and tetraphenyladamantane (NPN-3) as building blocks. To determine the interaction energies between COFs and CH_4_&H_2_, cluster models were generated for each COF to represent the gas binding sites (shown in Figure S2). DFT-level calculations provide a more accurate description of the electronic structure, particularly for interactions that govern binding energies, which are not fully captured by classical MD simulations. Electrostatic potential maps (ESPs) were then created for these optimized cluster models to visualize the electrostatic interactions between the adsorbents and adsorbates, as depicted in Figure 8. With the help of ESPs, the most favorable adsorption sites were identified by placing CH_4_ and H_2_ molecules in various positions relative to the COFs and optimizing the COF–gas pairs at the DFT level. Eight adsorption sites were determined for NPN-1, NPN-2, and NPN-3: aromatic phenyl rings (P1, P2, P3, P4, P7, and P8) and azodioxy connections (P5, P6) on the linkers. ESPs reveal that these eight regions are rich in electrons and function as effective electron-donor sites. The binding energies (Δ*E*_bind_) between the three COFs and the gases were calculated to assess the affinities of the gases for the COFs and are presented in Figure 8. While CH_4_ binds to three COFs with favorable interactions (negative values indicate attractive interactions) at eight regions determined, in most of the regions, H_2_ exhibits mostly nonfavorable or no interactions (positive values indicate repulsive interactions), which is attributed to the weaker polarizability of H_2_ (α = 0.787 Å^3^)^65^ compared to CH_4_ (α = 2.448 Å^3^).^65^ Thus, the average Δ*E*_bind_ values of H_2_ were calculated based on the attractive interactions.

As a representation, the interactions between CH_4_ and NPN-3 in eight identified adsorption sites are presented in Figure S12. In P1 and P2, CH_4_ forms C–H···π interactions with the center of masses of aromatic phenyl rings of tetraphenylmethane (NPN-1), tetraphenylsilane (NPN-2), and tetraphenyladamantane (NPN-3) scaffolds (Figure S12a,b) with similar Δ*E*_bind_ (7.91–8.64 kJ/mol, Figure 8), which are driven by the dispersion forces between two components. In P3, CH_4_ interacts with the center of mass of the phenyl ring, which is attached to azodioxy moieties as in P1 and P2, but slightly stronger (Δ*E*_bind_ = 8.61–9.17 kJ/mol) due to the more dispersed nature of the electrons of this fragment because of the electronegative azodioxy linkage (Figure S12c). In P4, in addition to the C–H···π interactions between CH_4_ and phenyl ring, a H-bond is observed between C–H···O-NNO (azodioxy) with bond lengths of 2.53, 2.55, and 2.65 Å and bond angles of 168.5°, 168.2°, and 160.7° for NPN-1, NPN-2, and NPN-3 (Figure S12d), respectively, which strengthen the adsorption of CH_4_ (Δ*E*_bind_ = 9.50–10.52 kJ/mol). CH_4_ is stabilized at the top face of the azodioxy linkage at P5 through a H-bond formed with an O atom with bond lengths of 2.66 and 2.51 Å in NPN-1 and NPN-2, with calculated Δ*E*_bind_ values of 7.93 and 9.14 kJ/mol, respectively. On the other hand, in NPN-3, two O atoms of azodioxy linkage are involved in H-bonds, with bond lengths of 2.68 and 2.72 Å (Figure S12e) presenting a Δ*E*_bind_ of 9.43 kJ/mol. When CH_4_ binds to the COFs at the P6 region, from the bottom face of the azodioxy linkage, two H-bonds are formed between CH_4_ and NPN-1/NPN-2/NPN-3 with bond lengths of 2.57 and 2.57 Å/2.61 and 2.57 Å/2.68 and 2.72 Å (Figure S12f), respectively, with Δ*E*_bind_ ranging between 8.23–10.12 kJ/mol. In P7 and P8, the interactions of CH_4_ with the aromatic phenyl rings of three COFs are mainly driven by the dispersion forces, as observed in P1 and P2 (Figure S12f,g) for which Δ*E*_bind_ ranges between 7.46 and 8.78 kJ/mol. These results point out that azodioxy linkages in these COFs enhance the binding affinity of CH_4_ molecules since they can form H-bonds.

As can be seen from Figure S13, in which the interactions between H_2_ and NPN-3 are displayed, H_2_ makes poor contacts with the COFs through weak van der Waals interactions in P1, P2, P3, P4, P7, and P8, with Δ*E*_bind_ < 1 kJ/mol (Figure S13a–d,g–h). Although weak H-bonds are observed in P5 and P6 with bond lengths of 2.48, 2.49, 2.44 Å and bond angles of 159.98°, 163.46°, 171.37° for NPN-1, NPN-2, NPN-3 (Figure S13e,f), respectively, the calculated Δ*E*_bind_ presented in Figure 8 indicate the presence of slightly repulsive interactions (Δ*E*_bind_ = 0.70–1.63 kJ/mol) due to the small and mobile nature of H_2_. Overall, P3 and P4 are the only possible binding sites for H_2_, which present negative Δ*E*_bind_, since these aromatic sites are more polarized due to the azodioxy linkages, as explained above.

The results indicate that CH_4_ binds to the three COFs with similar average Δ*E*_bind_ values of 8.55, 8.54, and 8.97 kJ/mol for NPN-1, NPN-2, and NPN-3, respectively (Figure 8). H_2_ also exhibits similar Δ*E*_bind_ values, excluding the positive quantities, with average values of 0.55, 0.27, and 0.86 kJ/mol for NPN-1, NPN-2, and NPN-3, respectively (Figure 8).

The weaker affinity of H_2_ for COFs compared to that of CH_4_ suggests that CH_4_ binds more strongly to the COFs, occupying all available adsorption sites and leaving H_2_ unbound within the pores. Thus, as a result of the cooperative effect between the smaller kinetic diameter of H_2_ and its poor adsorption strength, the passage of H_2_ molecules is facilitated through the membrane, while CH_4_ molecules are captured by the COF linkers. This behavior indicates that H_2_ is preferentially obtained at the end of the separation process, aligning with the results from the CGD-MD simulations. The H_2_/CH_4_ selectivities for NPN-1 and NPN-2 were calculated to be similar at 3.7 and 3.8, respectively, while the selectivity for NPN-3 was slightly higher at 6.1. This increased selectivity for NPN-3 can be attributed to the larger linker surface area of NPN-3 due to the bigger adamantane cage it bears (thus larger PLD) compared to that of NPN-1 and NPN-2, allowing for the binding of more CH_4_ molecules. As a result, a greater number of H_2_ molecules can pass through the membrane, enhancing the H_2_ selectivity of NPN-3.

We then used a crude transition state theory approach (66) to understand the diffusion barrier triggered by adsorption time or diffusion time in these COF membranes. Here, *k*(*T*) is the hopping rate of the gas molecules, Δ*E* is the gas binding energies of NPN-1, NPN-2, and NPN-3 calculated from DFT calculations, *k*_B_ is the Boltzmann constant (1.38 × 10^–23^ J/K), *h* is the Planck constant (6.63 × 10^–34^ J·K), *T* is the temperature (298 K), and *R* is the ideal gas constant (8.314 J/mol·K). Self-diffusion coefficients computed from EMD simulations of NPN-1, NPN-2, and NPN-3 were also used to compute diffusion time . Figure S14 compares the adsorption time versus diffusion time for these three COF membranes. As shown in Table S3, as the PLD decreases, the adsorption time and diffusion time become comparable. However, in NPN-3, these two times are more distinctly separated. This analysis may highlight the necessity of applying the NEMD method in cases where adsorption and diffusion times closely overlap. In our future work, we aim to focus on this aspect through detailed DFT calculations and provide a more comprehensive comparison.

## 4 Conclusions

Our results showed that the impact of pore size is pivotal in the computational assessment of COF membranes. Variations in the pore size influence adsorption and diffusion behaviors, directly affecting the gas permeation and selectivity of these materials. Understanding this relationship allows for optimized computational models, enhancing our ability to predict the performance of COF membranes and tailor their properties for gas separation applications. Our CGD-MD simulations consistently demonstrated that the investigated COF membranes are selective for H_2_ over CH_4_, in harmony with the results obtained from experimental measurements and DFT calculations. Conversely, the GCMC+EMD approach incorrectly indicated reverse selectivity for H_2_, especially for COFs with narrow pore dimensions. We finally proposed a relationship between the adsorption time and diffusion time, highlighting the critical role of selecting an appropriate simulation method.

Our findings suggest that for accurate prediction of membrane permselectivity in H_2_/CH_4_ separations, using the CGD-MD approach will be a good strategy. The equilibrium concentration profiles and minimal errors in *z*-density profiles underscore the robustness of the simulation methodology, paving the way for future experimental validation and potential applications of COF membranes in gas separation technologies.

## Supporting Information Available

The data set can be found on https://zenodo.org/ (DOI:10.5281/zenodo.14055573). The Supporting Information is available free of charge at https://pubs.acs.org/doi/10.1021/acsami.4c20420.

Structural properties of 13 COFs studied in this work (Table S1); number of CH_4_ and H_2_ molecules passed through the COF-300 membrane during a 20 ns simulation at room temperature and 1 bar (Figure S1); 3D representations of the frameworks (up) and cluster models generated for DFT calculations (down) for NPN-1 (a), NPN-2 (b), and NPN-3 (c) (Figure S2); concentration profiles for the ICR and OCR for COF membranes (Figures S3–S6); density profile of single-component CH_4_ (black) and single-component H_2_ (orange) as a function of *z* coordinate for COF-300 and COF-320 membranes (Figure S7); concentration profiles for ICR and OCR of COF-300 membrane obtained from NEMD simulations using DREIDING force field parameters (Figure S8); comparison of H_2_ permeabilities and H_2_/CH_4_ selectivities of the COF-300 membrane obtained from NEMD simulations using DREIDING and UFF force field parameters at 298 K and 1 bar (Table S2); density profile of the CH_4_ and H_2_ mixture as a function of the *z* coordinate for the COF-300 membrane during the production run (Figure S9); mean residence time profile of single-component and mixture of CH_4_ (black) and H_2_ (orange) in the *z*-direction for COF-300 (Figure S10); concentration profiles for the ICR and OCR of NPN-3 at 10 bar (Figure S11); interactions of CH_4_ with NPN-3 at identified regions P1 (a), P2 (b), P3 (c), P4 (d), P5 (e), P6 (f), P7 (g), and P8 (h) (Figure S12); interactions of H_2_ with NPN-3 at identified regions P1 (a), P2 (b), P3 (c), P4 (d), P5 (e), P6 (f), P7 (g), and P8 (h) (Figure S13); adsorption time for H_2_ and CH_4_ as a function of their diffusion time within NPN-1, NPN-2, and NPN-3 membranes (Figure S14); and diffusion and adsorption times for H_2_ and CH_4_ in three COFs (NPN-1, NPN-2, NPN-3) with their respective PLDs (Table S3) (PDF)
