# Supplementary material for: Harnessing Pore Size in COF Membranes: A Concentration Gradient-Driven Molecular Dynamics Study on Enhanced H2/CH4 Separation
Source: ACS Appl Mater Interfaces. 2025 Mar 1;17(10):15372–84. doi: 10.1021/acsami.4c20420 (PMC11912198; doi:10.1021/acsami.4c20420)
Supplement: Supplementary file 1 — am4c20420_si_001.pdf [file am4c20420_si_001.pdf]

Supporting Information

for

Harnessing Pore Size in COF Membranes: A Concentration Gradient-Driven Molecular Dynamics (CGD-MD) Study on Enhanced H_2/CH_4 Separation

Parivash Jamshidi Ghaleh[a], Zeynep Pinar Haslak[b], Merdan Batyrow[a], Ilknur Erucar[b*]

[a]Department of Mechanical Engineering, Faculty of Engineering, Ozyegin University, Cekmekoy, 34794, Istanbul, Turkey.
[b]Department of Natural and Mathematical Sciences, Faculty of Engineering, Ozyegin University, Cekmekoy, 34794, Istanbul, Turkey.

* Email: ilknur.erucar@ozyegin.edu.tr

Table S1. The structural properties of 13 COFs studied in this work. The table includes the system size (replication), unit cell source from CURATED-COFs[1] database (original or optimized), pore limiting diameter (PLD), the largest cavity diameter (LCD), accessible surface area (ASA) with a probe radius of 1.86 Å, and the porosity.

COF NAME	Chemical Structure	System size (replication)	Structure database	PLD (Å)	LCD (Å)	ASA (m^2/g)	Porosity (Volume Fraction)
**COF-300**	imine linked tetraphenyl methane and phenyl polymer network[2]	2x2x5	Original	9.26	9.36	3254.4	0.73
**COF-320**	imine linked tetraphenyl methane and biphenyl polymer network[3]	2x2x7	Original	8.29	8.45	1804.3	0.63
**COF-303**	imine linked tetraphenyl methane and phenyl polymer network[4]	2x2x7	Optimized	8.31	8.60	1827.1	0.61
**COF-921**	abenzo[1,2-d:4,5-d']bis(thiazole) linked 1,3,6,8-tetraphenylpyrene polymer network[5]	2x2x7	Optimized	17.28	17.77	1797.8	0.70
**3D-Por-COF**	tetraphenyl methane and tetrakis(4-methylbenzene)-porphyrin polymer network[6]	1x2x2	Optimized	13.84	16.47	6082.4	0.84
**NPN-1**	Azodioxy linked tetraphenyl methane polymer network[7]	4x4x7	Original	4.11	5.37	906.2	0.51
**NPN-2**	Azodioxy linked tetraphenyl silane polymer network[7]	4x4x7	Original	4.19	5.27	1074.6	0.54
**NPN-3**	Azodioxy linked tetraphenyl adamantane polymer network[7]	3x3x7	Original	5.44	6.14	942.6	0.49
**TPE-COF-I**	imine linked tetraphenyl ethene polymer network[8]	2x3x5	Original	7.17	7.82	1817.2	0.60
**COF-IM AA**	2,5-dimethoxybenzene linked 3,5-bis(4-(imidazol-1-yl)quinolin-6-yl)-1,1'-biphenyl polymer network[9]	2x2x6	Original	24.17	24.46	1553.9	0.70
**DMTA-TPB2**	tetraphenyl methane and 1,4-dimethoxy-2,5-dimethylbenzene polymer network[10]	2x2x7	Optimized	18.90	19.62	1956.5	0.73
**TfpBDH**	pyromellitic diimide linked 1,3,5-tris(4-methyl phenyl)benzene polymer network[11]	2x2x13	Original	36.16	36.32	2098.4	0.82
**PCOF-2**	3,3',5,5'-tetrakis(4-metylphenyl)bimesityl linked 5, 10, 15, 20-tetra(4-aminobiphenyl)porphyrin polymer network[12]	1x2x2	Original	26.28	28.08	6867.5	0.92

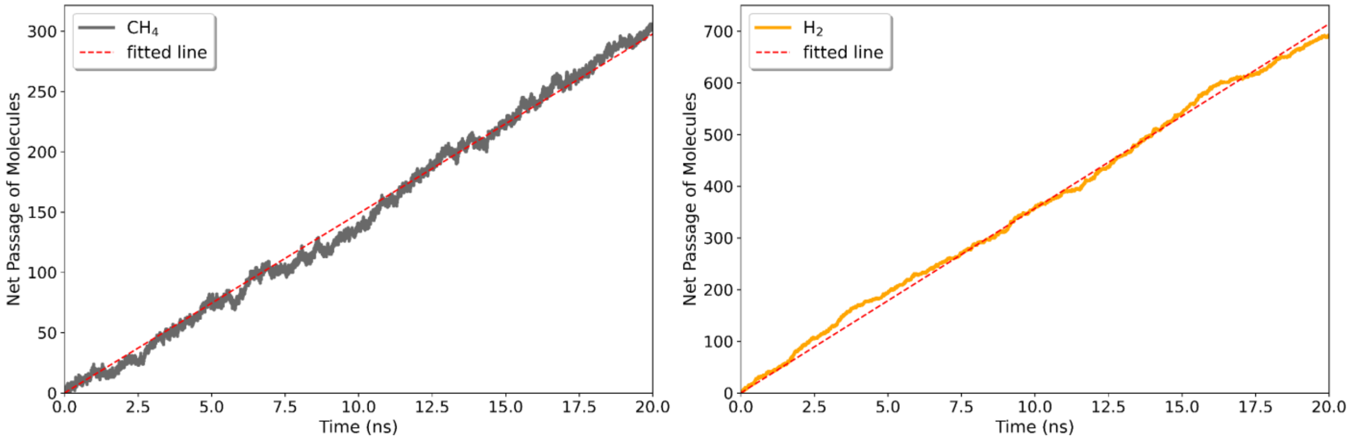

Figure S1. The number of CH_4 and H_2 molecules passed through COF-300 membrane during a 20 ns simulation at room temperature and 1bar.

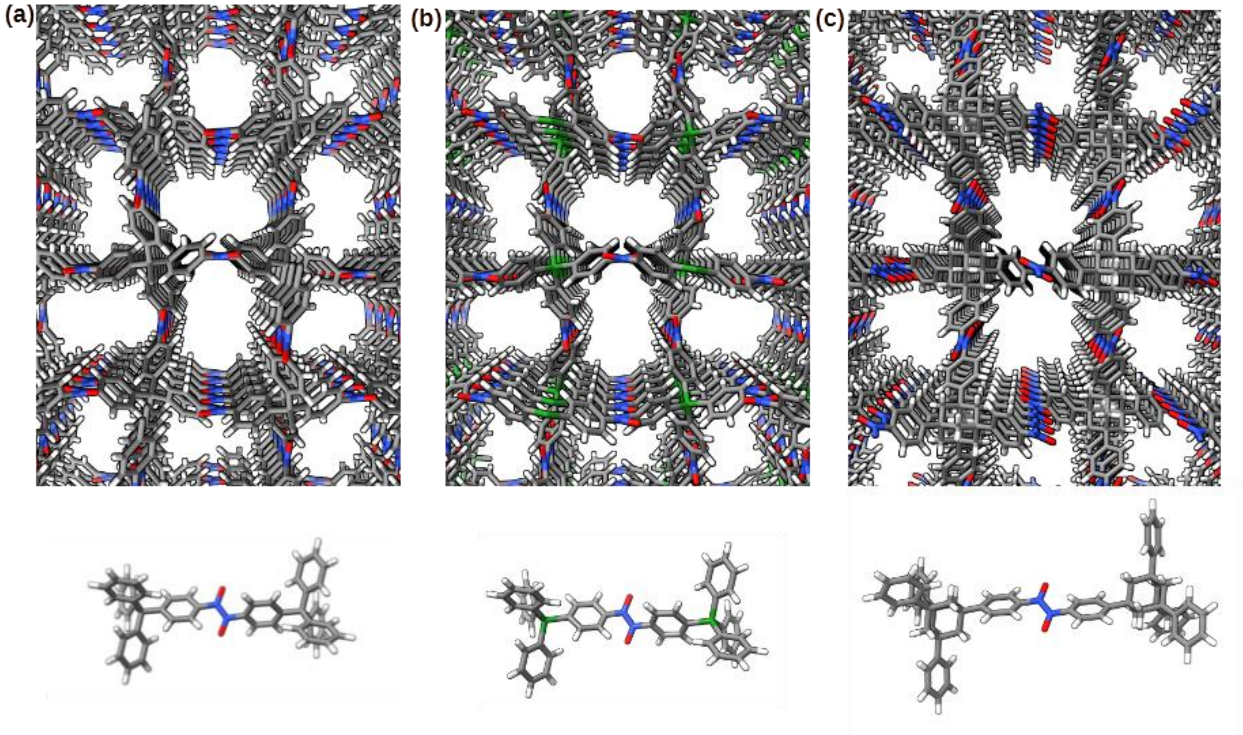

Figure S2. 3D representations of the frameworks (up) and cluster models generated for DFT calculations (down) for NPN-1 (a), NPN-2 (b) and NPN-3 (c). C, H, O, N and Si atoms are represented with gray, white, red, blue and green colors, respectively.

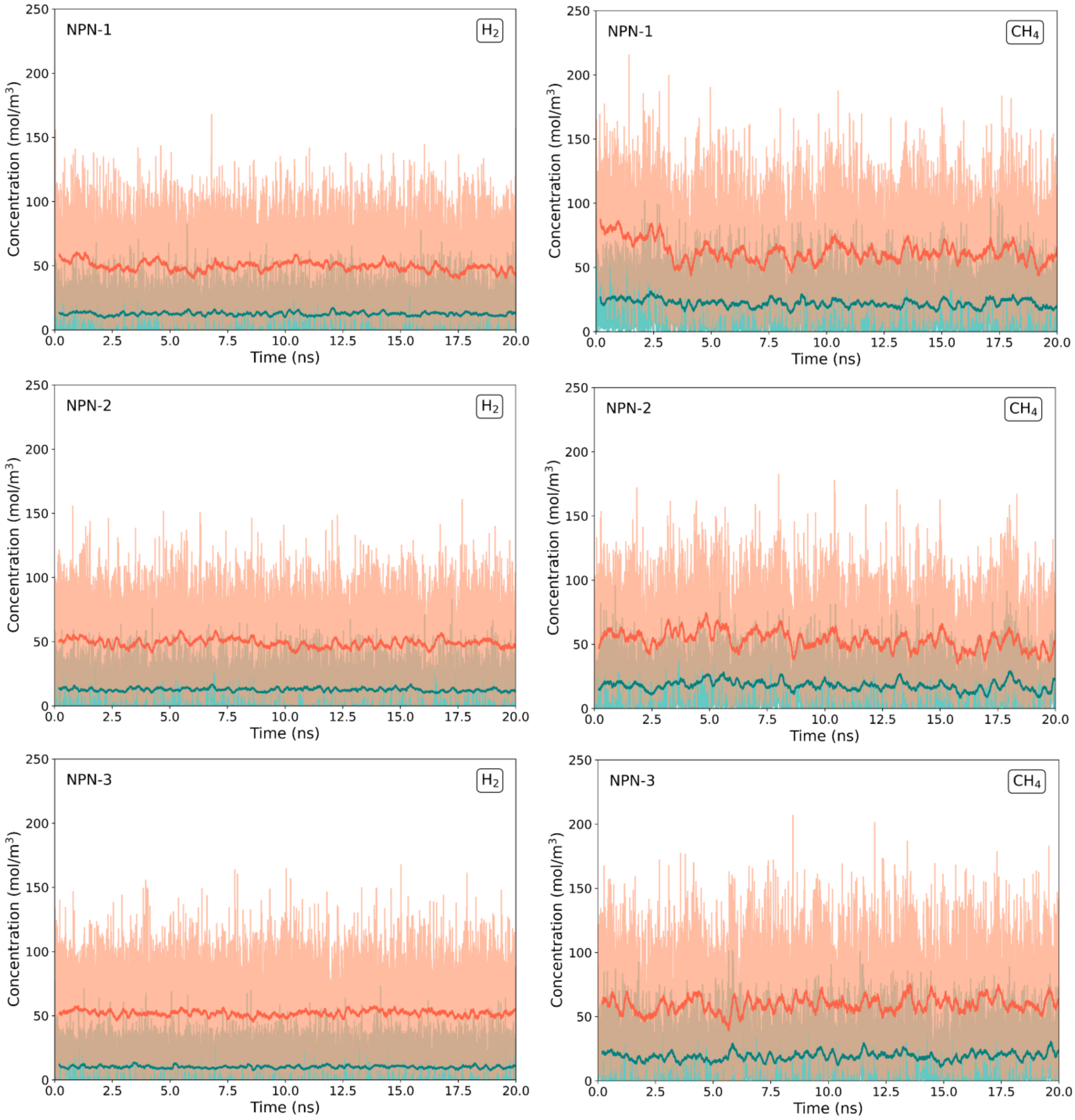

Figure S3. The concentration profiles for ICR and OCR of NPN-1, NPN-2, NPN-3 during production runs of NEMD simulations. Instantaneous concentrations of ICR and OCR are shown in light orange and light green, respectively, while their smoothed averages, computed with a 0.2 ns smoothing time, are depicted by solid lines of corresponding colors.

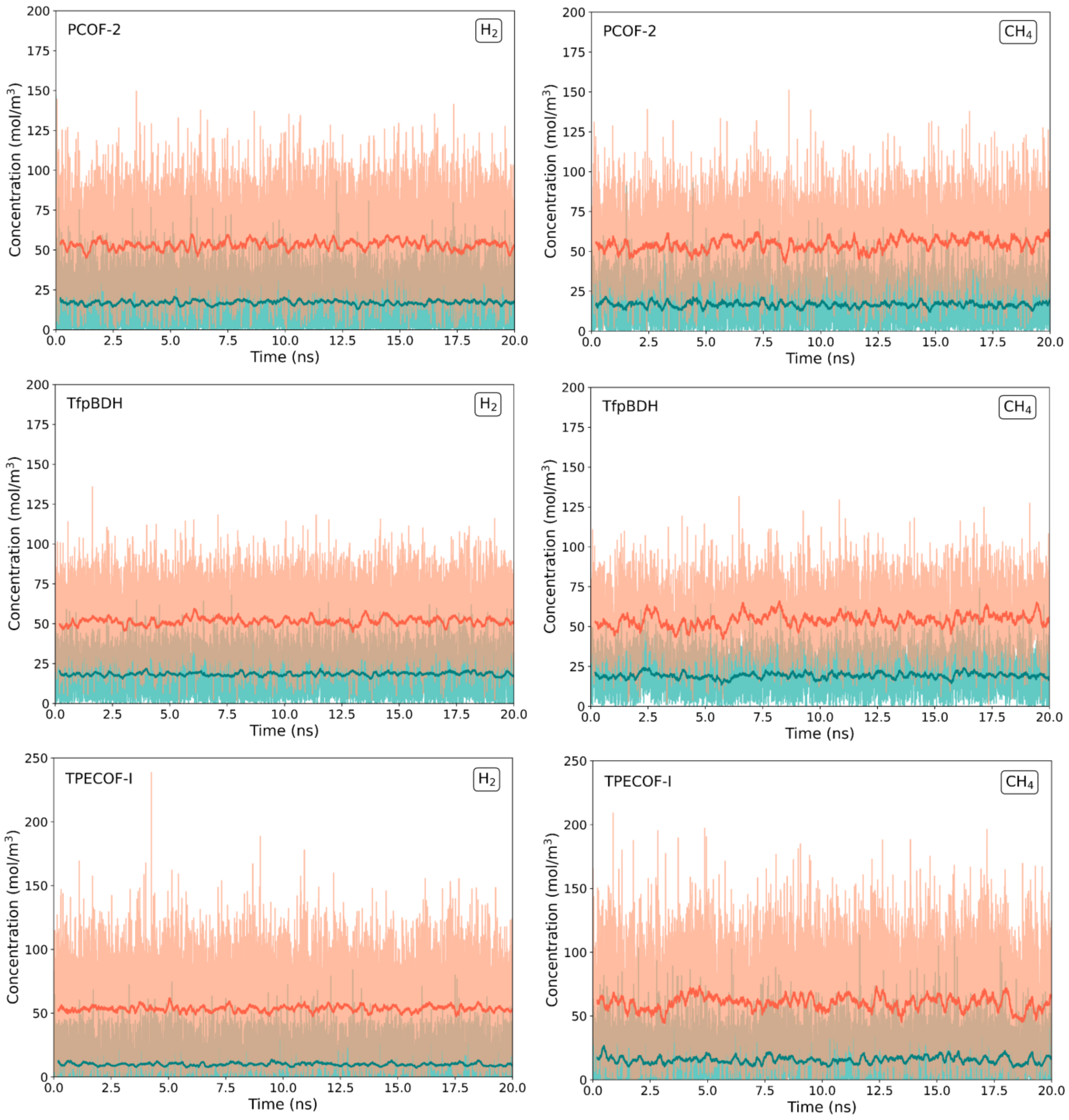

Figure S4. The concentration profiles for ICR and OCR of PCOF-2, TfpBDH, TPE-COF-I during production runs of NEMD simulations.

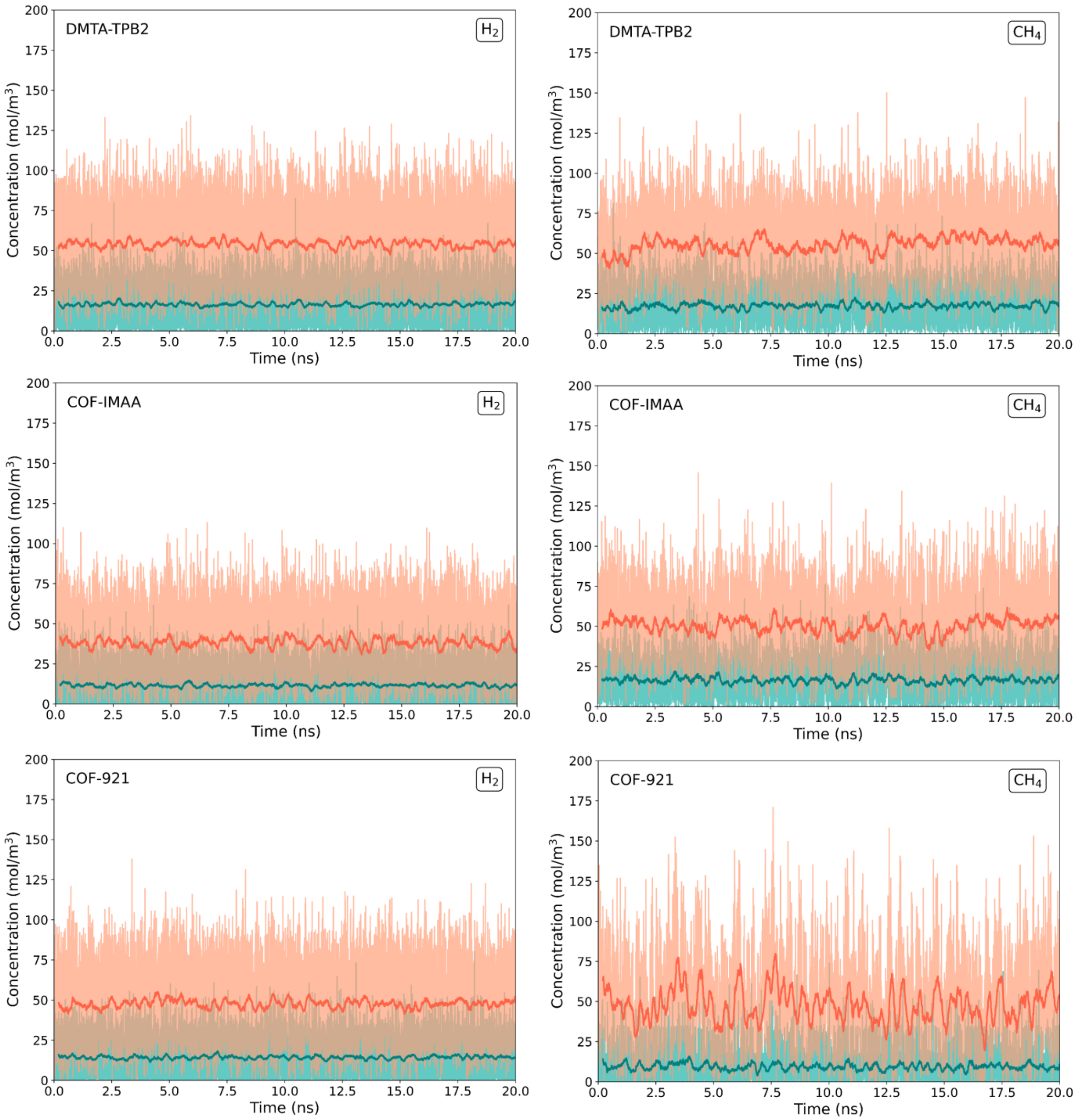

Figure S5. The concentration profiles for ICR and OCR of DMTA-TPB2, COF-IM AA, COF-921 during production runs of NEMD simulations.

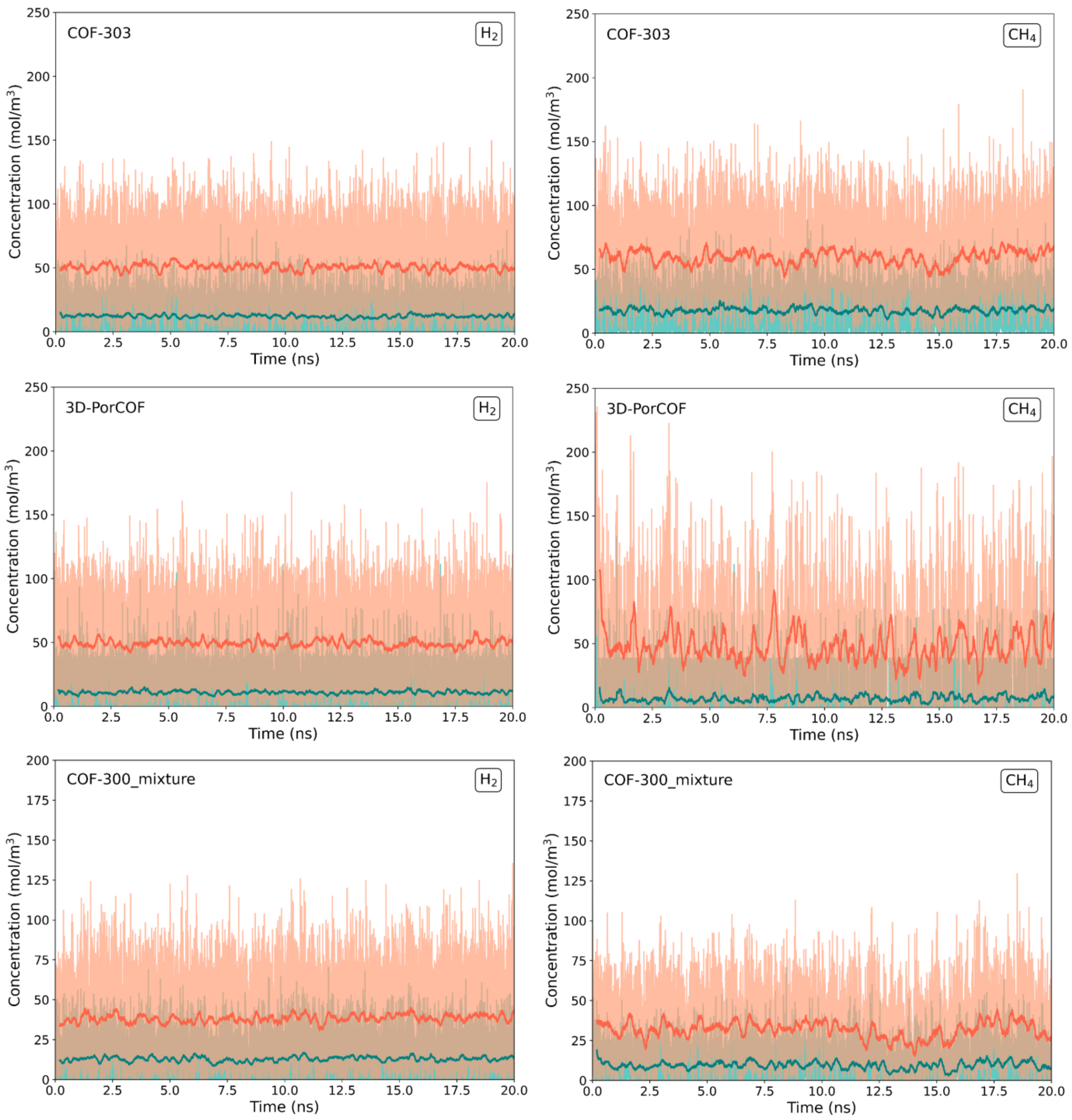

Figure S6. The concentration profiles for ICR and OCR of COF-303, 3D-PorCOF, COF-300 (mixture gas) during production runs of NEMD simulations.

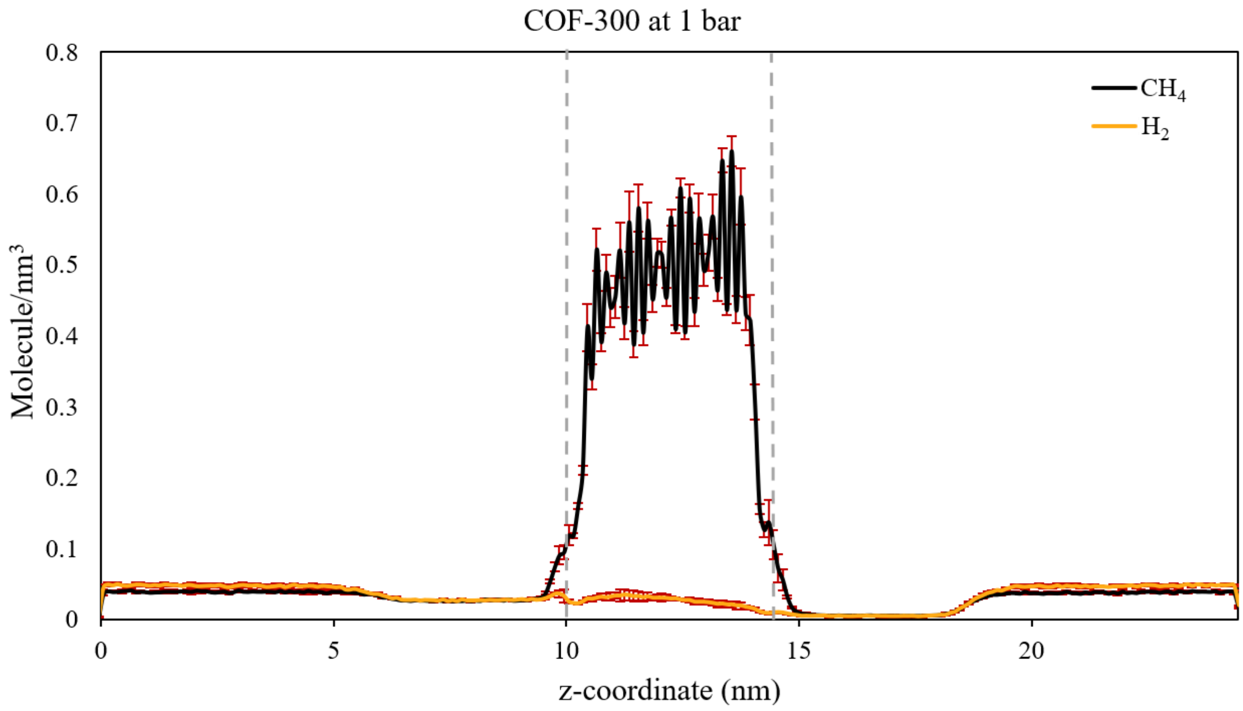

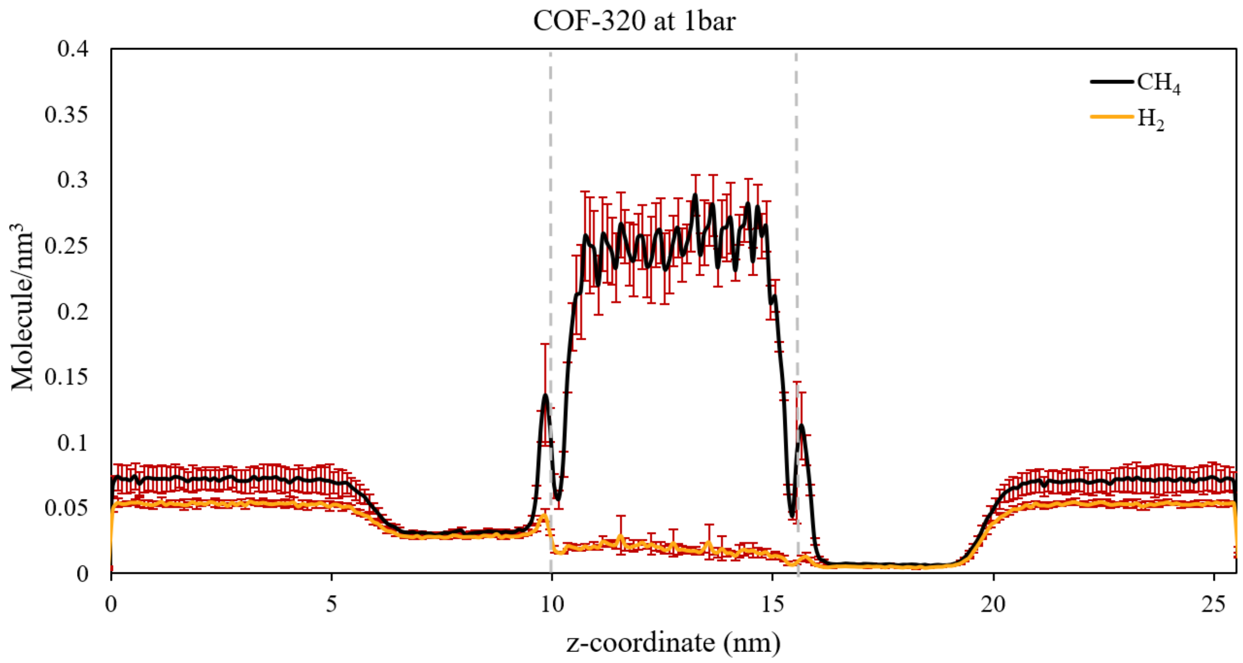

Figure S7. The density profile of single-component CH_4 (black) and single-component H_2 (orange) as a function of z coordinate for COF-300 and COF-320 membranes. The dashed vertical lines represent the membrane region.

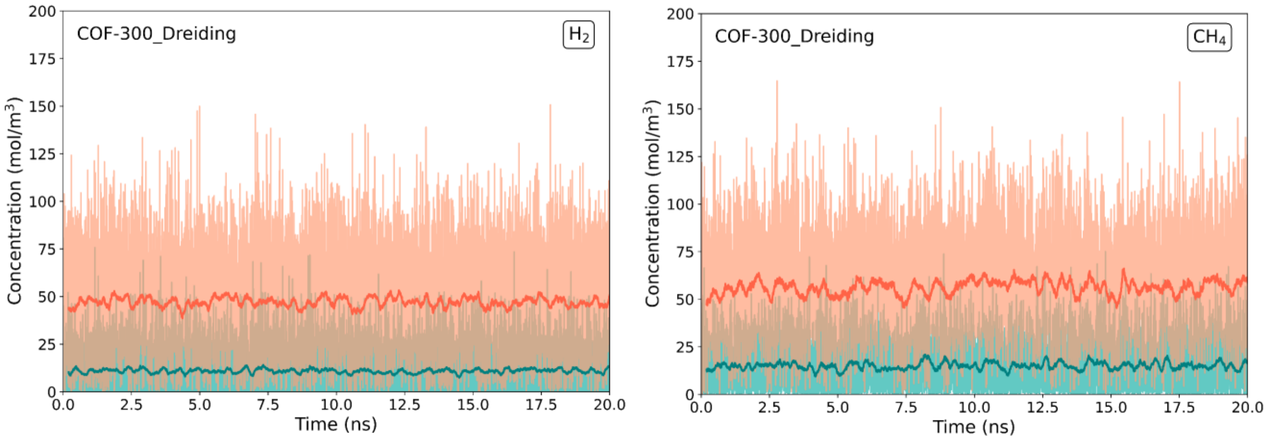

Figure S8. The concentration profiles for ICR and OCR of COF-300 membrane obtained from NEMD simulations using DREIDING force field parameters.

Table S2. Comparison of H_2 permeabilities and H_2/CH_4 Selectivities of the COF-300 membrane obtained from NEMD simulations using DREIDING and UFF force field parameters at 298 K and 1 bar.

Force field	H_2 Permeability x 10^5 (Barrer)	H_2/CH_4 Selectivity
**UFF**	2.60 ± 0.14	1.97 ± 0.12
**Dreiding**	2.49 ± 0.12	2.25 ± 0.02

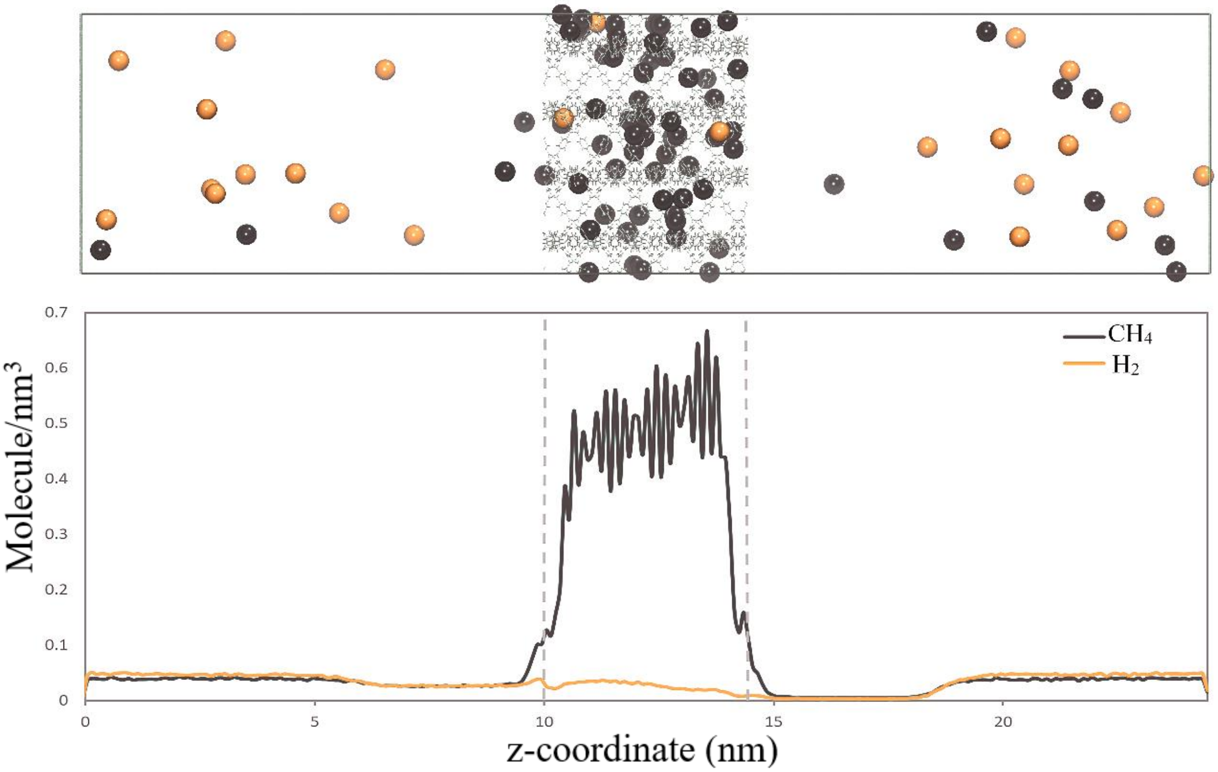

Figure S9. The density profile of CH_4 and H_2 mixture as a function of the z coordinate for COF-300 membrane during the production run.

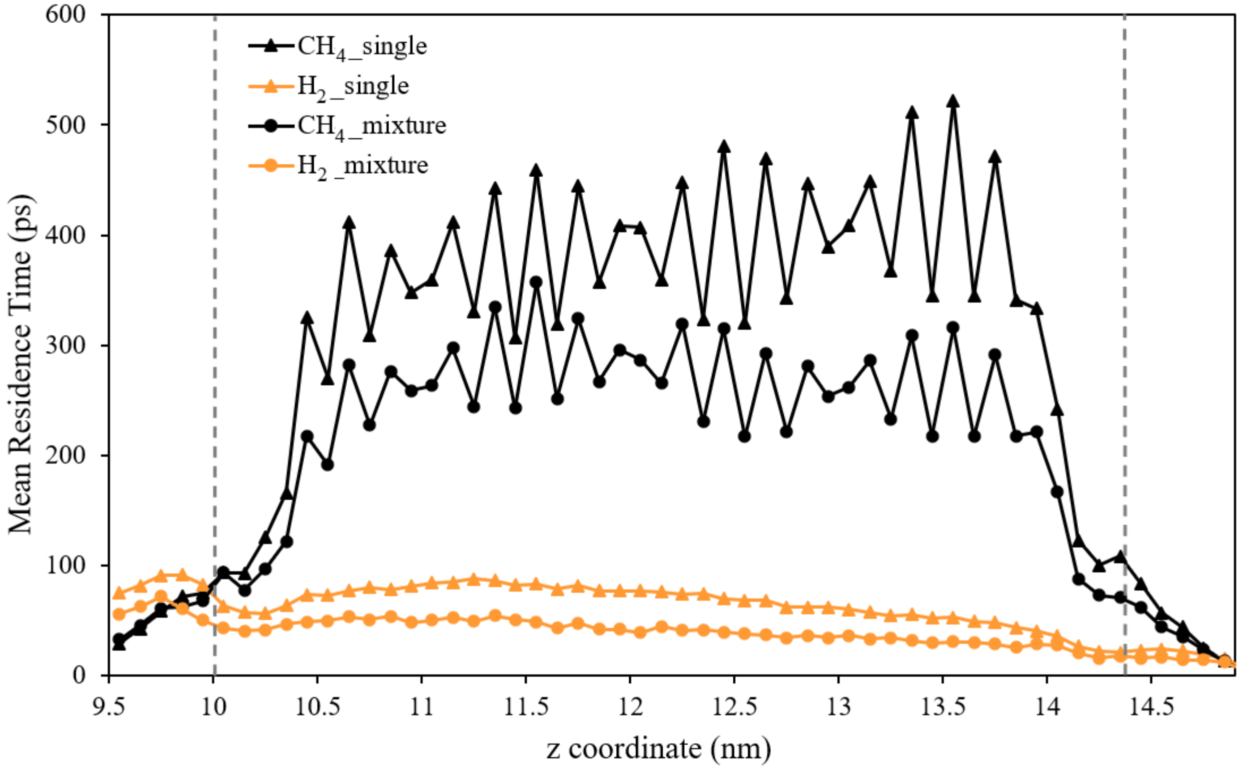

Figure S10. The mean residence time profile of single-component and mixture of CH_4 (black) and H_2 (orange) in the z-direction for COF-300. Each point represents the mean residence time of the molecules during the production simulation within a bin width of 0.1 nm. The dashed lines indicate the positions of the membrane surfaces.

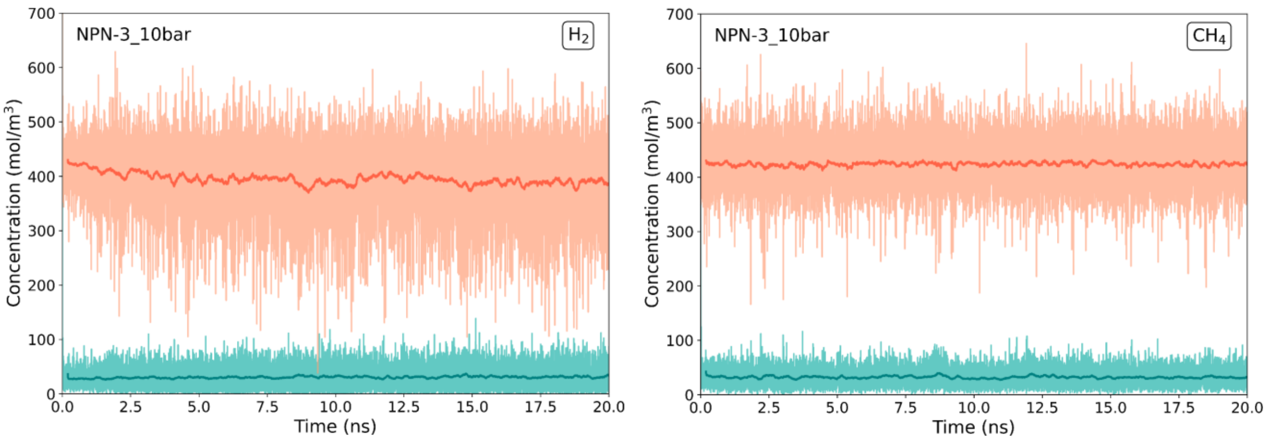

Figure S11. The concentration profiles for ICR and OCR of NPN-3 at 10 bar during production runs.

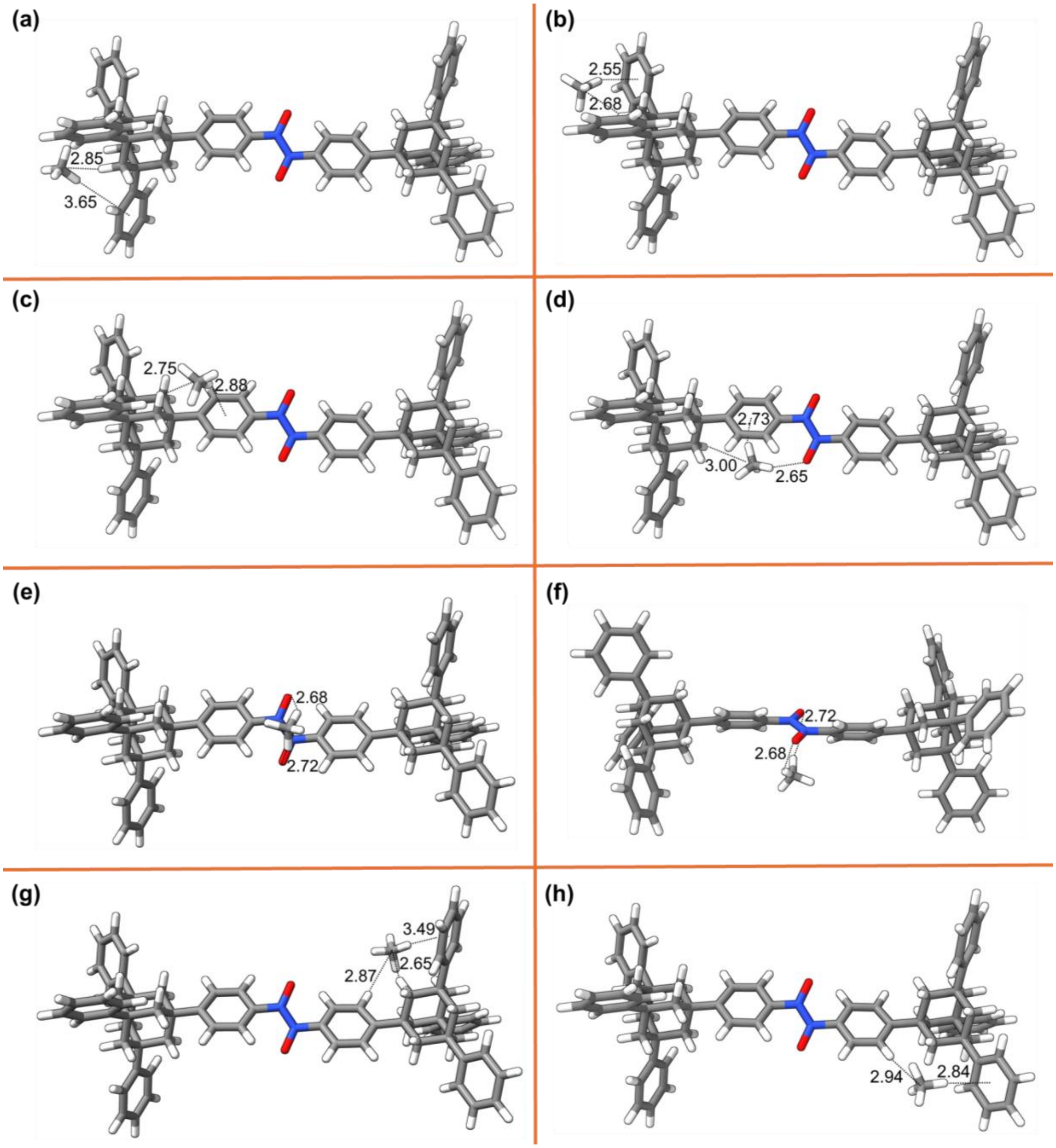

Figure S12. Interactions of CH_4 with NPN-3 at identified regions P1 (a), P2 (b), P3 (c), P4 (d), P5 (e), P6 (f), P7 (g) and P8 (h). Distances are given in Å. White: hydrogen, gray: carbon, red: oxygen, blue: nitrogen.

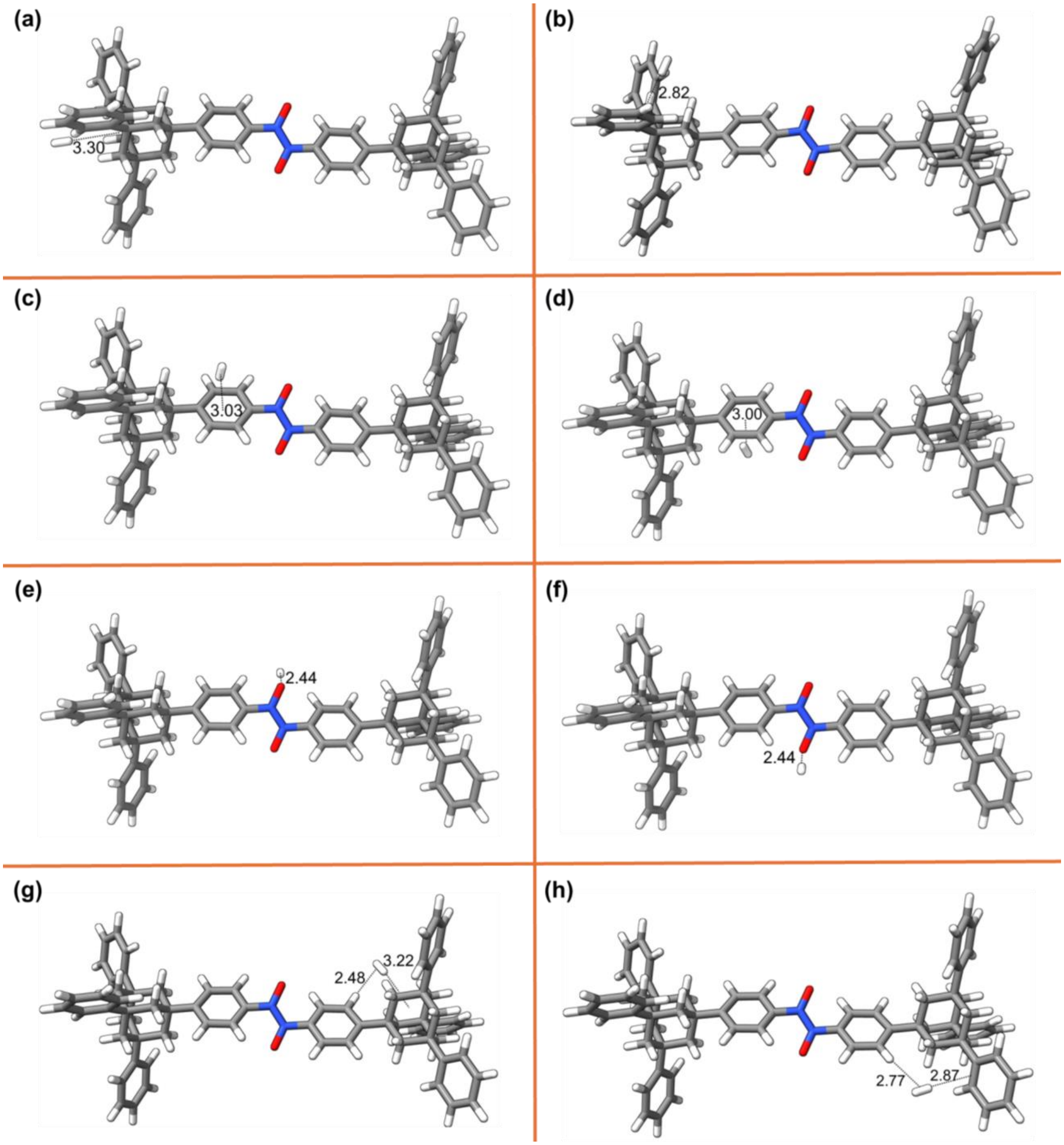

Figure S13. Interactions of H_2 with NPN-3 at identified regions P1 (a), P2 (b), P3 (c), P4 (d), P5 (e), P6 (f), P7 (g) and P8 (h). Distances are given in Å. White: hydrogen, gray: carbon, red: oxygen, blue: nitrogen.

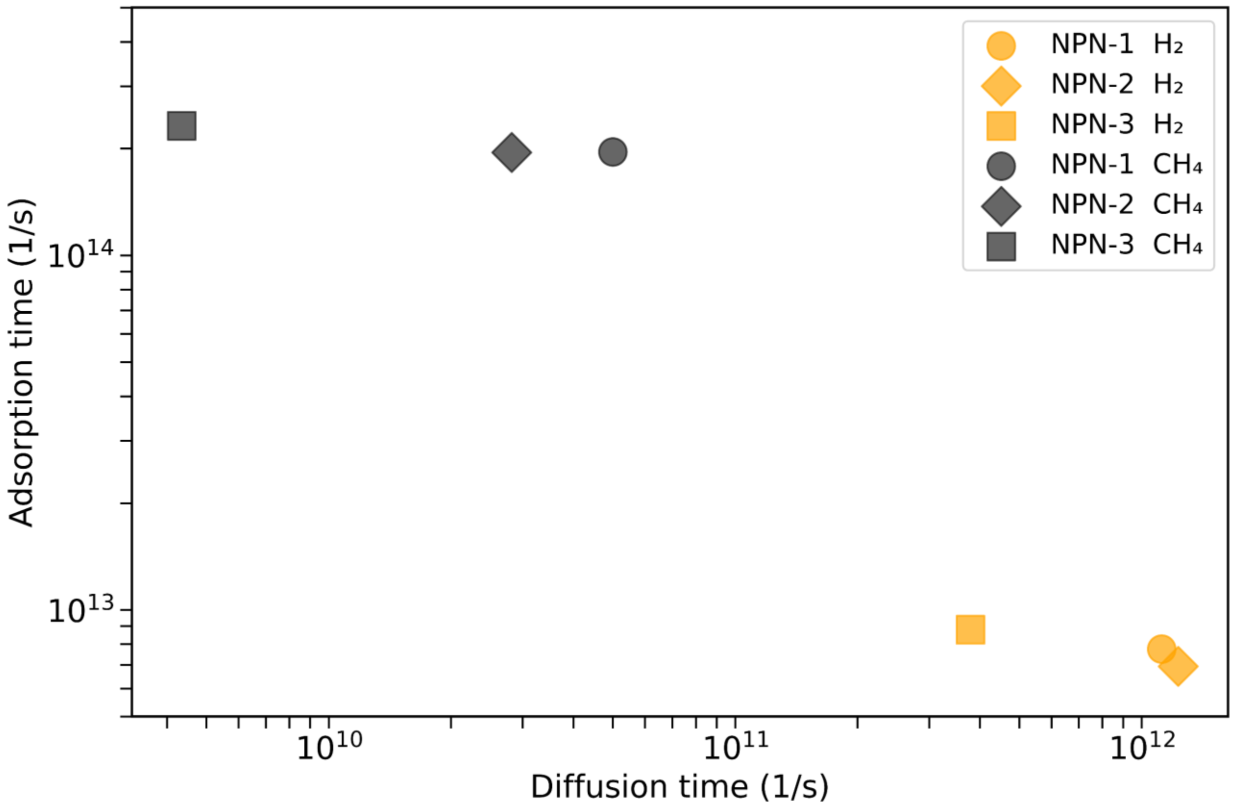

Figure S14. The adsorption time for H_2 and CH_4 as a function of their diffusion time within NPN-1, NPN-2, and NPN-3 membranes.

Table S3. Diffusion and adsorption times for H_2 and CH_4 in three COFs (NPN-1, NPN-2, NPN-3) with their respective pore limiting diameters (PLDs).

COFs NAME	PLD(Å)	H_2		CH_4	
		Diffusion time ($\times10^{12}$ 1/s)	Adsorption time ($\times10^{12}$ 1/s)	Diffusion time ($\times10^{12}$ 1/s)	Adsorption time ($\times10^{12}$ 1/s)
**NPN-1**	4.11	1.120	7.75	0.050	195.78
**NPN-2**	4.19	1.230	6.92	0.028	194.99
**NPN-3**	5.44	0.379	8.79	0.004	231.95

References:

(1) Ongari, D.; Yakutovich, A. V.; Talirz, L.; Smit, B. Building a consistent and reproducible database for adsorption evaluation in covalent–organic frameworks. *ACS central science* **2019**, *5* (10), 1663-1675.

(2) Uribe-Romo, F. J.; Hunt, J. R.; Furukawa, H.; Klock, C.; O'Keeffe, M.; Yaghi, O. M. A crystalline imine-linked 3-D porous covalent organic framework. *J. Am. Chem. Soc.* **2009**, *131* (13), 4570-4571.

(3) Zhang, Y.-B.; Su, J.; Furukawa, H.; Yun, Y.; Gándara, F.; Duong, A.; Zou, X.; Yaghi, O. M. Single-crystal structure of a covalent organic framework. *J. Am. Chem. Soc.* **2013**, *135* (44), 16336-16339.
(4) Ma, T.; Kapustin, E. A.; Yin, S. X.; Liang, L.; Zhou, Z.; Niu, J.; Li, L.-H.; Wang, Y.; Su, J.; Li, J. Single-crystal x-ray diffraction structures of covalent organic frameworks. *Science* **2018**, *361* (6397), 48-52.
(5) Waller, P. J.; AlFaraj, Y. S.; Diercks, C. S.; Jarenwattananon, N. N.; Yaghi, O. M. Conversion of imine to oxazole and thiazole linkages in covalent organic frameworks. *J. Am. Chem. Soc.* **2018**, *140* (29), 9099-9103.
(6) Lin, G.; Ding, H.; Chen, R.; Peng, Z.; Wang, B.; Wang, C. 3D porphyrin-based covalent organic frameworks. *J. Am. Chem. Soc.* **2017**, *139* (25), 8705-8709.
(7) Beaudoin, D.; Maris, T.; Wuest, J. D. Constructing monocrystalline covalent organic networks by polymerization. *Nat. Chem.* **2013**, *5* (10), 830-834.
(8) Gao, Q.; Li, X.; Ning, G.-H.; Xu, H.-S.; Liu, C.; Tian, B.; Tang, W.; Loh, K. P. Covalent organic framework with frustrated bonding network for enhanced carbon dioxide storage. *Chem. Mater.* **2018**, *30* (5), 1762-1768.
(9) Yao, B.-J.; Wu, W.-X.; Ding, L.-G.; Dong, Y.-B. Sulfonic acid and ionic liquid functionalized covalent organic framework for efficient catalysis of the Biginelli reaction. *J. Org. Chem.* **2021**, *86* (3), 3024-3032.
(10) Zhang, J.; Han, X.; Wu, X.; Liu, Y.; Cui, Y. Multivariate chiral covalent organic frameworks with controlled crystallinity and stability for asymmetric catalysis. *J. Am. Chem. Soc.* **2017**, *139* (24), 8277-8285.
(11) Das, G.; Biswal, B. P.; Kandambeth, S.; Venkatesh, V.; Kaur, G.; Addicoat, M.; Heine, T.; Verma, S.; Banerjee, R. Chemical sensing in two dimensional porous covalent organic nanosheets. *Chem. Sci.* **2015**, *6* (7), 3931-3939.
(12) Liu, Y.; Yan, X.; Li, T.; Zhang, W.-D.; Fu, Q.-T.; Lu, H.-S.; Wang, X.; Gu, Z.-G. Three-dimensional porphyrin-based covalent organic frameworks with tetrahedral building blocks for single-site catalysis. *New J. Chem.* **2019**, *43* (43), 16907-16914.
